# Biocompatible ionized air alleviates rat osteoarthritis by modulating polarization from M1 to M2 macrophages

**DOI:** 10.1038/s41598-024-83198-6

**Published:** 2024-12-30

**Authors:** Haoran Yu, Chengbiao Ding, Zhongyao Hu, Qi Liu, Xuesong Gu, Junyan He, Yiqun Yan, Shenrui Yu, Lin Gao, Wendan Cheng, Zhengwei Wu, Juehua Jing

**Affiliations:** 1 Department of Orthopedics, The Second Affiliated Hospital of Anhui Medical University, Hefei, 230000 China; 2 Institute of Orthopedics, Research Center for Translational Medicine, The Second Affiliated Hospital of Anhui Medical University, Hefei, 230000 China; 3 Department of Rehabilitation Medicine, The Second Affiliated Hospital of Anhui Medical University, Hefei, 230000 China; 4 The Second Clinical Medical College of Anhui Medical University, Hefei, 230000 China; 5 The First Clinical Medical College of Anhui Medical University, Hefei, 230000 China; 6 School of Nuclear Science and Technology, University of Science and Technology of China, Hefei, 230000 China

**Keywords:** Biocompatible ionized air, Osteoarthritis, Macrophages, Osteoarthritis, Plasma physics

## Abstract

The imbalance in the proportion of M1/M2 macrophage polarization is a crucial contributor to the persistent progression of osteoarthritis (OA). This study aimed to evaluate the effects of low-dose biocompatible ionized air (BIA) on macrophage polarization and its subsequent chondroprotective effects, thereby validating the potential of BIA in slowing the progression of OA. In vitro experiments demonstrated that BIA modulates the polarization of M1 macrophages toward the M2 phenotype via the ROS-mediated STAT6 pathway. This shift reduces the expression of pro-inflammatory mediators while increasing the expression of anti-inflammatory mediators and pro-chondrogenic factors, leading to an improved microenvironment surrounding chondrocytes. The direct benefits of this improved microenvironment include enhanced chondrocyte viability, inhibition of apoptosis, and reduced degradation of the extracellular matrix. In vivo studies in rats showed that BIA inhibited M1 macrophage infiltration in the synovium, upregulated the proportion of M2 macrophages, alleviated cartilage degeneration, and delayed OA progression. This gas-based regulatory strategy may open new avenues for the treatment of OA.

**Supplementary Information:**

The online version contains supplementary material available at 10.1038/s41598-024-83198-6.

## Introduction

Osteoarthritis (OA), a prevalent chronic joint disease, is characterized by structural changes in joint cartilage, remodeling of the underlying bone, the development of osteophytes, and inflammation of the synovium, often leading to joint pain, swelling, stiffness, and significant functional limitations^1–3^. Currently, the treatment of OA predominantly relies on oral medications; the widely used pharmacological treatments can only mitigate clinical symptoms and alleviate pain caused by OA^4,5^ . However, they cannot repair damaged joints or slow OA progression^6,7^. Additionally, patients may experience potential side effects from these medications, such as gastrointestinal ulcers induced by nonsteroidal anti-inflammatory drugs (NSAIDs)^2,8^. Therefore, further exploring more efficient and safe therapeutic strategies for OA is imperative.

Mounting research indicates that synovial macrophages play a crucial role in the initiation and progression of OA^9–11^. In the physiological synovial membrane, pro-inflammatory M1 macrophages and anti-inflammatory M2 macrophages are maintained in a dynamic balance. However, under the inflammatory stimulation of OA, quiescent M0 macrophages polarize towards M1 macrophages, leading to an imbalance in the M1/M2 macrophage ratio and promoting the progression of OA^12–14^. Therefore, regulating macrophage polarization to restore the M1/M2 balance may be an effective therapeutic strategy for OA. Currently, most studies focus on using drugs to regulate macrophage polarization for treating OA^15,16^. However, current pharmacotherapy for joint diseases has inherent limitations. The penetrative nature of drugs makes it difficult to localize their action to the affected joint, which can result in unintended harm to non-target organs^17^. Therefore, there is an urgent need for a means to target and regulate macrophage polarization in affected joints.

Biocompatible ionized air (BIA) is a non-thermal plasma that utilizes air as the ionized gas, enabling various biomedical applications below 40 °C. This property gives it excellent biocompatibility. Plasma, an ionized gas, contains reactive oxygen species (ROS), electromagnetic components, light, and ultraviolet rays^18–20^. Among these, ROS, including singlet oxygen, ozone, and hydroxyl radicals, are considered one of the main effector molecules^21–23^. These ROS play a dual role: at high doses and long durations, they induce cell senescence and apoptosis, while at low doses and short durations, they stimulate cell migration, proliferation, and repair^24–27^. In recent years, plasma has evolved from a niche in physics to a prominent discipline in the biomedical field, with applications in diseases such as cancer, skin conditions, and trauma^28–30^. This study not only found that BIA can regulate the polarization of M1 macrophages to M2 macrophages^31^, but also revealed in early investigations into the mechanisms of BIA-mediated macrophage polarization that the activation level of STAT6 was significantly higher compared to critical proteins in other pathways. Previous research has suggested that ROS can induce M2 macrophage polarization by modulating STAT6 activation^32^, and in this study, ROS is identified as one of the main effector molecules of BIA. Therefore, this study hypothesizes that BIA regulates M1-to-M2 macrophage polarization via the ROS-mediated STAT6 pathway, offering further insights into how BIA influences macrophage polarization. Given that the production technology of BIA allows for targeted delivery to diseased joints, BIA has the potential to become an efficient and safe targeted therapeutic approach for OA.

This study respectively developed BIA devices for in vivo and in vitro experiments. Initially, the study investigated BIA’s positive effects on macrophage polarization and chondrogenesis-related gene expression. Additionally, potential mechanisms by which BIA regulates M1-to-M2 macrophage polarization were explored. Subsequently, the chondroprotective effects of co-culturing BIA-treated M1 macrophages with chondrocytes were evaluated. Finally, the therapeutic value of BIA for OA was confirmed through intra-articular intervention in OA rats.

## Results

### Determination of BIA components and device operating temperature

**Fig. 1:**
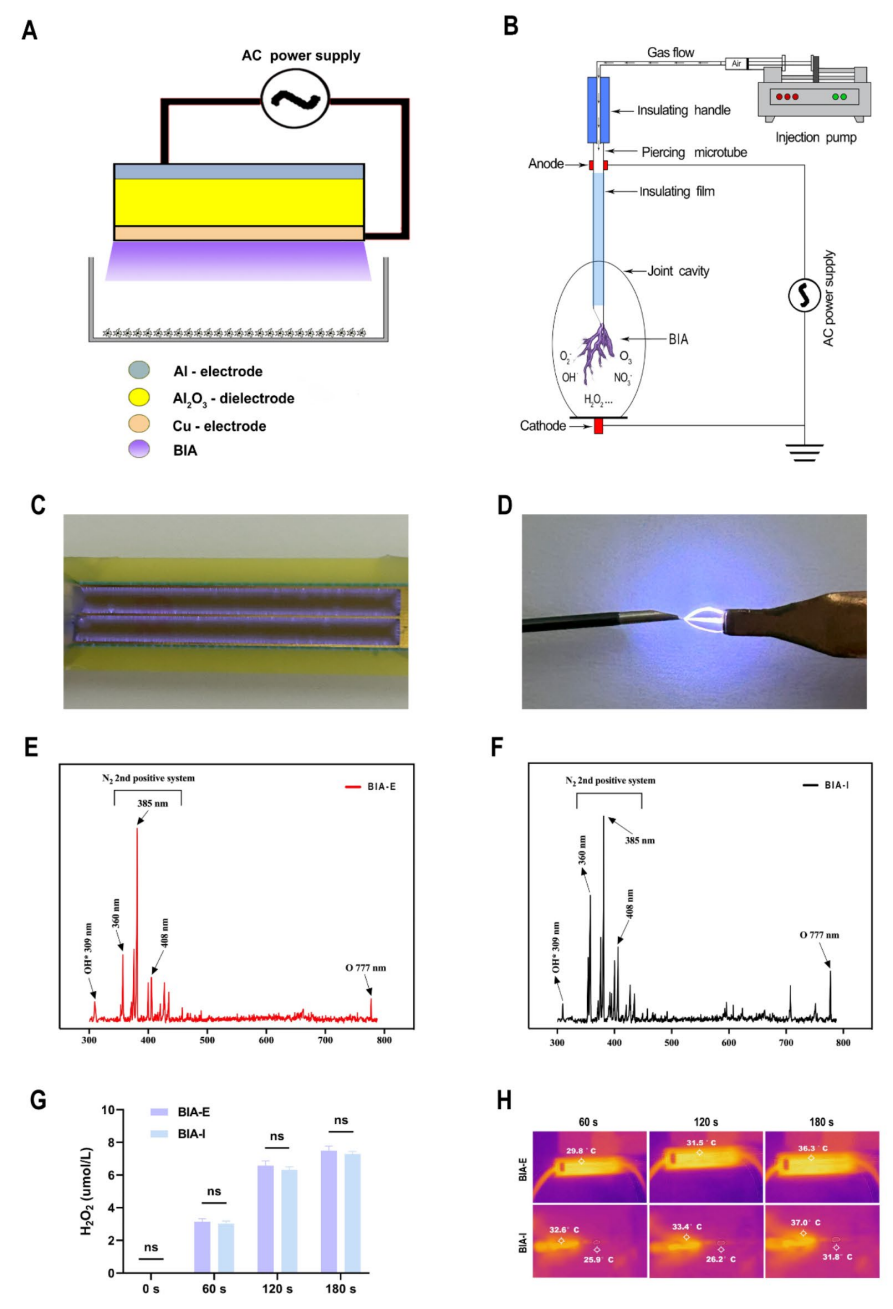
Schematic of BIA devices and plasma physicochemical parameters. (**A**) Operating schematic of BIA-E device. (**B**) Operating schematic of BIA-I device. (**C**) Physical discharge diagram of BIA-E device. (**D**) Physical discharge diagram of BIA-I device. (**E**)**–**(**F**) Emission spectra of BIA-E and BIA-I devices between 300 and 800 nm. (**G**) Comparison of H_2_O_2_ levels generated by BIA-E and BIA-I devices at different operating times. (**H**) Working temperature of BIA-I and BIA-E devices over time. ‘ns’ indicates no significant difference between the two groups.

Spectral analysis was performed on both devices to validate the consistency of BIA components and doses. Results showed high consistency in the composition produced by both BIA devices (Fig. 1E and F). H_2_O_2_ concentration in DMEM after plasma treatment was measured to estimate the ROS dosage of the BIA devices. The results demonstrate a gradual increase in ROS dosage with extended BIA treatment duration, displaying a time-dependent pattern. Furthermore, there was no significant difference in ROS dosage for the same treatment duration between the two devices (Fig. 1G), suggesting that both BIA devices generate consistent ROS levels under operational conditions. Temperature measurements (Fig. 1H) indicate that, for BIA-E, as discharge time increases from 60 to 180 s, the DBD panel’s temperature rises from 29.8 °C to 36.3 °C. Similarly, for BIA-I, as discharge time increases from 60 to 180 s, the arc column’s temperature rises from 25.9 °C to 31.8 °C, and the dielectric layer’s temperature increases from 32.6 °C to 37.0 °C. These findings indicate that both BIA devices operate below 40 °C, ensuring the safety of biological tissues during experimentation.

### Effects of BIA on intracellular ROS levels, cellular viability, and polarization shift in M1 macrophages

**Fig. 2:**
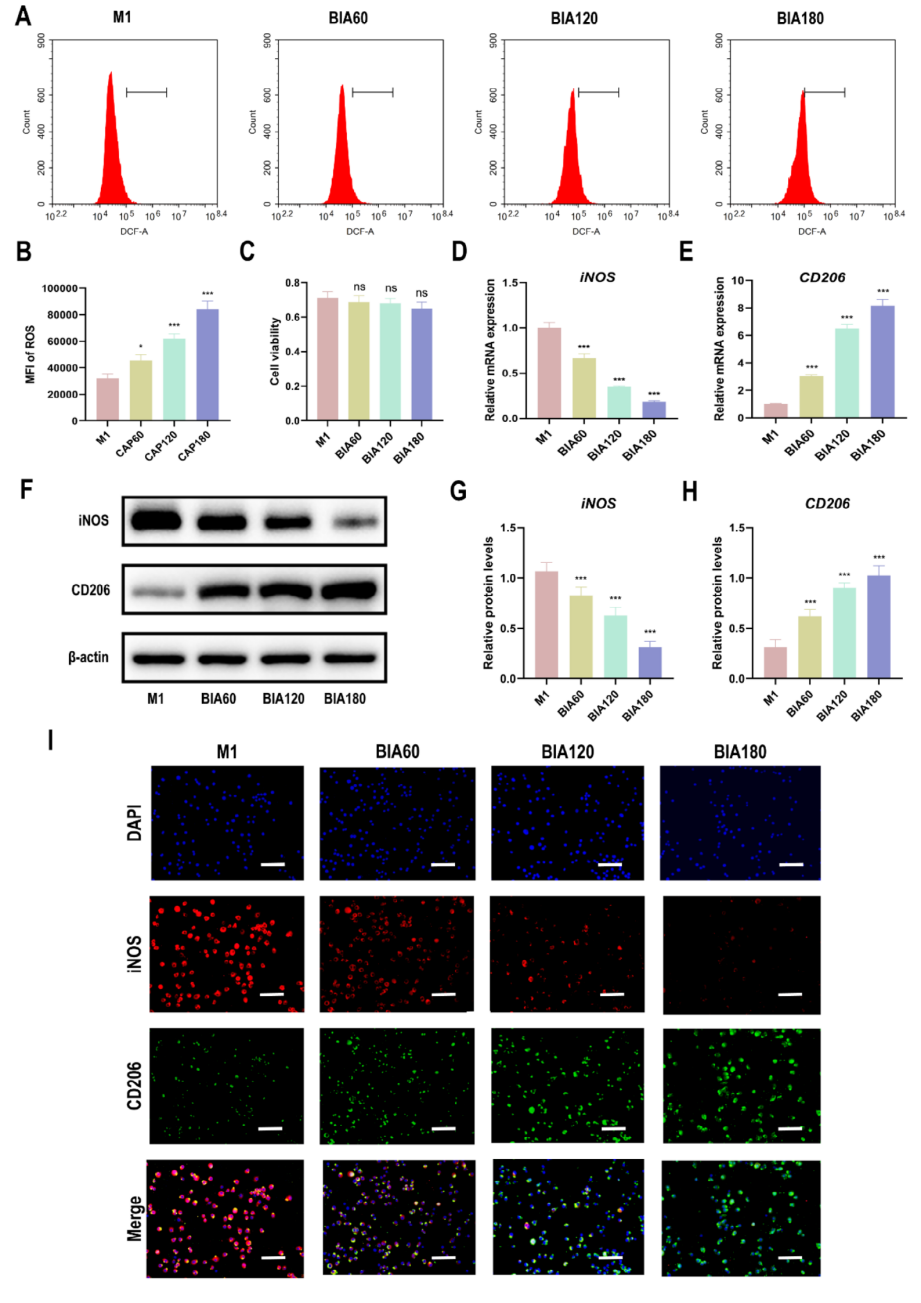
The effect of BIA on ROS levels, cellular viability, and polarization of M1 macrophages. (**A**), (**B**) DCFH-DA assay to evaluate BIA-induced changes in intracellular ROS levels in M1 macrophages. **(C)** CCK-8 assay to evaluate the effect of different BIA intervention durations on M1 macrophage viability. (**D**)–(**E**) qRT-PCR assay to evaluate gene expression levels of macrophage polarization markers (iNOS for M1 macrophages and CD206 for M2 macrophages) in M1 macrophages after different durations of BIA treatment. (**F**)–(**H**) Western blot to detect protein expression levels of macrophage polarization markers in M1 macrophages after different durations of BIA treatment. **(I)** Immunofluorescence assay to display representative fluorescence images of iNOS and CD206 in M1 macrophages after different durations of BIA treatment, with DAPI staining cell nuclei, scale bar = 50 μm. N = 3. *Significant difference to the M1 group: ***p < 0.001. Abbreviations: BIA: Biocompatible ionized air; ROS: reactive oxygen species; DCFH-DA: 2',7'- dichlorofluorescin diacetate assay; CCK-8: Cell Counting Kit-8; qRT-PCR: quantitative reverse transcription polymerase chain reaction; iNOS: inducible nitric oxide synthase; CD206: Cluster of Differentiation 206; DAPI: 4',6-diamidino-2-phenylindole.

As ROS is the primary effector of BIA, changes in intracellular ROS levels following BIA intervention at 60 s, 120 s, and 180 s in M1 macrophages were investigated. The results revealed that, compared to M1 macrophages without BIA treatment, intracellular ROS levels significantly increased after BIA intervention (Fig. 2A and B). Furthermore, with increasing BIA exposure time, intracellular ROS levels in M1 macrophages gradually rose time-dependent. BIA’s impact on M1 macrophage viability was also assessed, and the results demonstrated that 180 s of BIA treatment did not impair M1 macrophage viability (Fig. 2C). Additionally, BIA intervention for 180 s did not significantly affect chondrocyte viability or apoptosis (Fig. S1). Thus, the ROS levels in BIA are at non-harmful, low doses.

The expression of iNOS (M1 macrophage marker) and CD206 (M2 macrophage marker) was assessed using qRT-PCR and western blot (WB) analysis. BIA downregulated iNOS expression at both the gene (Fig. 2D, E) and protein levels (Fig. 2F–H), while upregulating CD206 expression. The shift from M1 to M2 macrophages became more pronounced with more prolonged BIA exposure, following a time-dependent trend similar to the increase in intracellular ROS levels. Furthermore, IF analysis further confirmed that BIA downregulates iNOS expression in M1 macrophages while concurrently upregulating CD206 expression (Fig. 2I). These findings suggest that BIA can regulate the polarization of M1 macrophages toward the M2 phenotype.

### BIA regulates the polarization of M1 macrophages towards M2 macrophages through the ROS-mediated STAT6 pathway

Analysis indicates that M1 macrophages increasingly polarize toward the M2 phenotype as intracellular ROS levels increase, suggesting a close association between BIA-generated ROS and macrophage polarization. This study found that STAT6 was significantly activated in BIA-treated M1 macrophages compared to other critical proteins from different pathways, including p-AKT, p-NF-κB p65, and Cleaved-Notch 1 (Fig. S2). BIA treatment of M1 macrophages was performed for 0 s and 180 s to further investigate the underlying mechanisms, followed by a 24-h incubation.

**Fig. 3:**
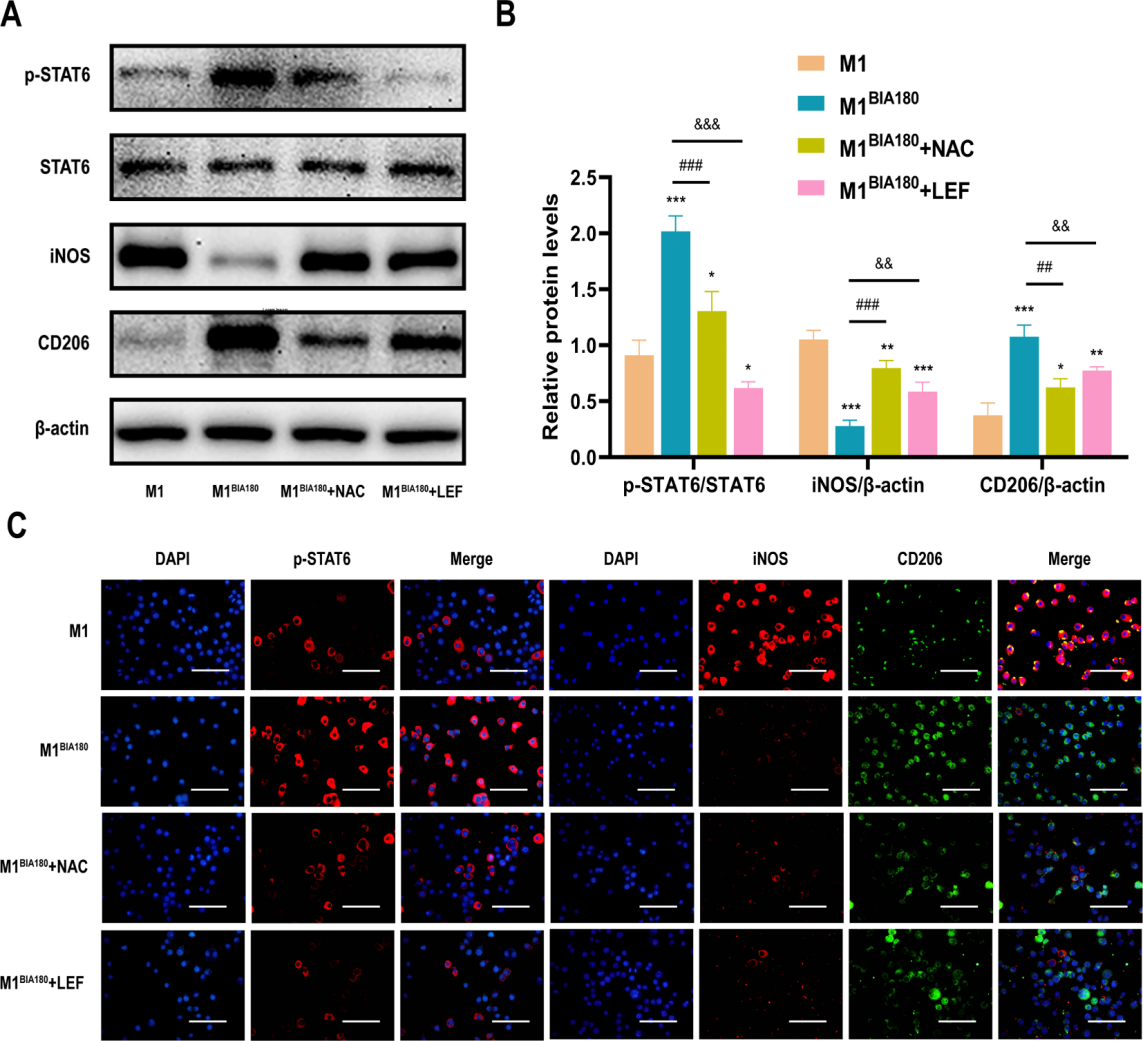
BIA regulates M1 macrophage polarization to M2 macrophages through the ROS-mediated STAT6 pathway. (**A**) Western blot analysis of p-STAT6, iNOS, and CD206 expression levels in macrophages from different groups after BIA intervention. (**B**) Quantitative analysis of p-STAT6, iNOS, and CD206 expression levels in macrophages. (**C**) Immunofluorescence assay to visualize representative fluorescence images of p-STAT6 and corresponding macrophage polarization markers iNOS and CD206 in M1 macrophages after different intervention conditions, with DAPI staining cell nuclei. Scale bar = 20 μm. N = 3. *Significant difference compared to the M1 group: *p < 0.05, **p < 0.01, ***p < 0.001. ^#^Significant difference compared to the M1^BIA180^ group: ^##^p < 0.01, ^###^p < 0.001. ^&^Significant difference compared to the M1^BIA180^ group: ^&&^p < 0.01, ^&&&^p < 0.001. Abbreviations: BIA: Biocompatible ionized air; ROS: reactive oxygen species; STAT6: Signal transducer and activator of transcription 6; qRT-PCR: quantitative reverse transcription polymerase chain reaction; iNOS: inducible nitric oxide synthase; CD206: Cluster of Differentiation 206; DAPI: 4',6-diamidino-2-phenylindole; NAC: N-Acetyl-L-cysteine; LEF: Leflunomide.

The Western blot (WB) results (Fig. 3A and B) showed a significant increase in phosphorylated STAT6 levels after BIA treatment. At the same time, iNOS (M1 marker) expression decreased, while CD206 (M2 marker) expression increased. M1 macrophages were then pretreated with NAC or LEF, followed by BIA treatment for 180 s and an additional 24-h cultivation. The results (Fig. 3A and B) indicate that, compared to the M1^BIA180^ group, p-STAT6 expression significantly decreased in macrophages pretreated with NAC and LEF. CD206 expression decreased, while iNOS expression increased, indicating reduced M1-to-M2 macrophage polarization. The IF assay results were consistent with these findings (Fig. 3C).

In summary, BIA treatment promotes STAT6 phosphorylation. Pre-treatment with NAC and LEF reduces BIA-induced STAT6 phosphorylation, decreasing M1-to-M2 macrophage polarization. These results indicate that ROS is the primary effector molecule of BIA, regulating M1-to-M2 macrophage polarization via the STAT6 pathway. The results (Fig. 3A–C) also show that, compared to the M1 group, NAC pre-treatment to neutralize ROS slightly increased p-STAT6 levels in the M1^BIA180^ + NAC group. Similarly, iNOS expression slightly decreased, while CD206 expression increased. After LEF treatment, p-STAT6 expression decreased, but the M1^BIA180^ + LEF group still slightly reduced iNOS and increased CD206 expression.

### M1 macrophage modulation into M2 macrophages by BIA exhibits anti-inflammatory and chondrogenic functions

**Fig. 4:**
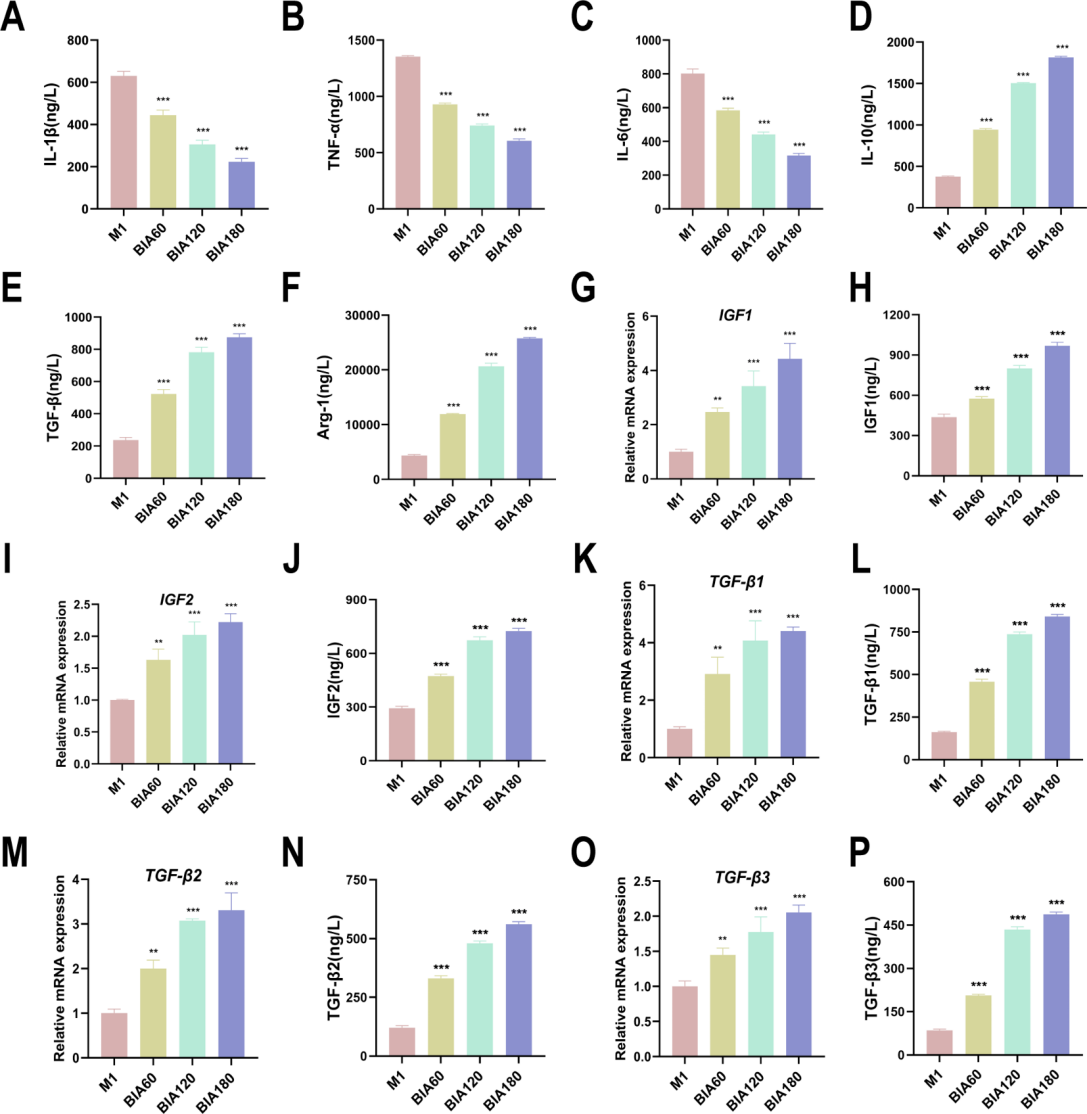
Functional changes of M1 macrophages into M2 macrophages induced by BIA. (**A**)–(**C**) ELISA evaluation of pro-inflammatory cytokines IL-1β, IL-6, and TNF-α expression levels in M1 macrophages after BIA intervention. (**D**)–(**F**) ELISA evaluation of anti-inflammatory cytokines IL-10, TGF-β, and Arg-1 expression levels in M1 macrophages after BIA intervention. (**G**)–(**P**) Expression levels of pro-chondrogenic factors IGF1, IGF2, TGF-β1, TGF-β2, and TGF-β3 in M1 macrophages after BIA intervention. N = 3. *Significant difference compared to the M1 group: **p < 0.01, ***p < 0.001. Abbreviations: BIA: Biocompatible ionized air; IL-1β: Interleukin-1β; IL-6: Interleukin-6; TNF-α: Tumor necrosis factor α; IL-10: Interleukin-10; TGF-β: Transforming growth factor β; Arg-1: Arginase-1; IGF1: Insulin-like growth factor; TGF-β1: Transforming growth factor β1.

To investigate the functional changes following BIA-induced M1-to-M2 macrophage polarization, ELISA was used to measure secreted pro-inflammatory and anti-inflammatory mediator levels in the culture medium of each M1 macrophage group. The results showed that BIA time-dependently decreased pro-inflammatory mediators IL-1β, IL-6, and TNF-α (Fig. 4A, B, C), while increasing anti-inflammatory mediators IL-10, TGF-β, and Arg-1 (Fig. 4D, E, F). Pro-chondrogenic factor expression in M2 macrophages helps establish a microenvironment conducive to chondrogenesis^16^. Thus, qRT-PCR was used to examine pro-chondrogenic gene expression in macrophages after BIA treatment. The results demonstrated that BIA upregulated the expression of pro-chondrogenic genes *IGF1*, *IGF2*, *TGF-β1*, *TGF-β2*, and *TGF-β3* in a time-dependent manner (Fig. 4G, I, K, M, O). ELISA further confirmed that BIA also increased the levels of secreted proteins IGF1, IGF2, TGF-β1, TGF-β2, and TGF-β3 in a time-dependent manner (Fig. 4H, J, L, N, P).

**Fig. 5:**
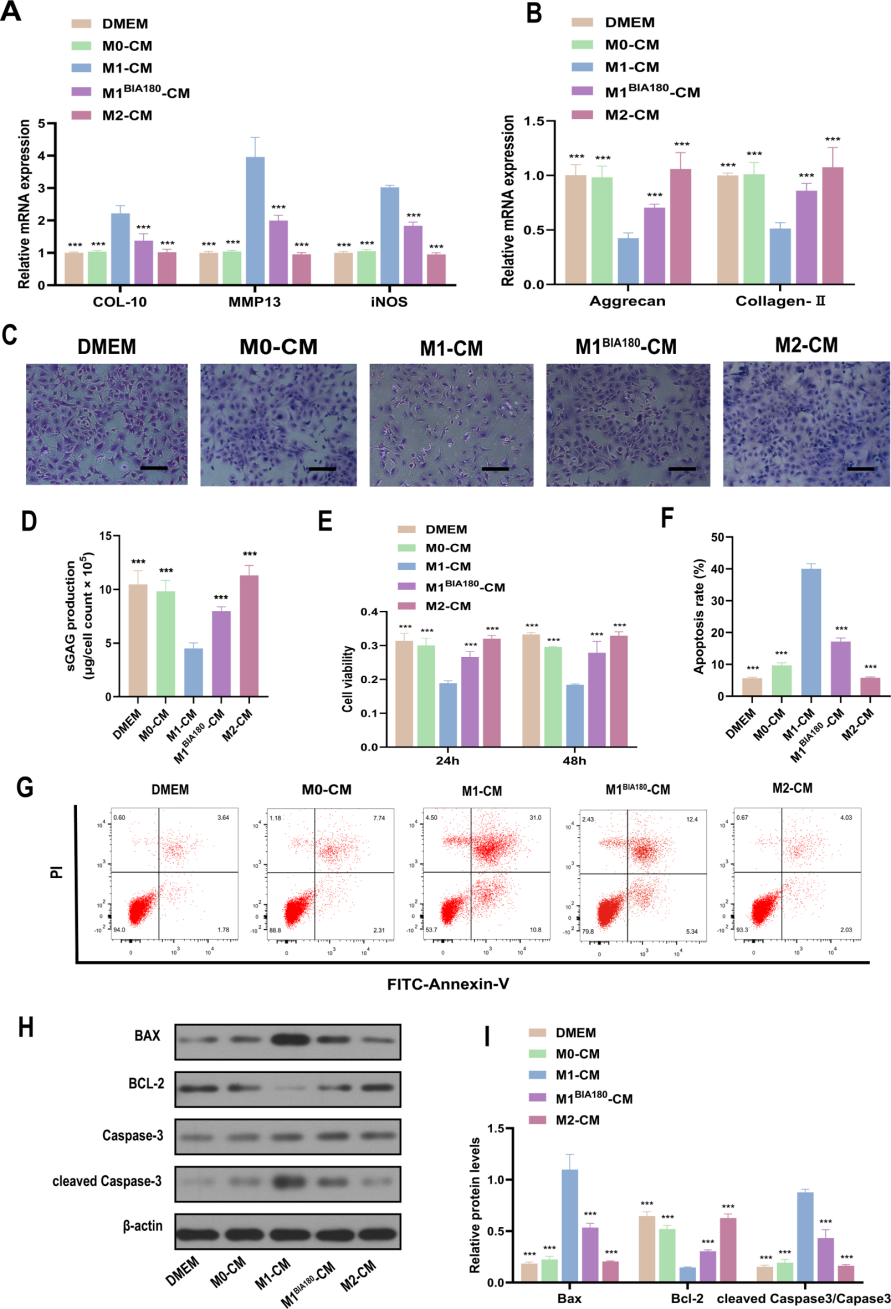
Different macrophage-CM co-culture with chondrocytes. (**A**) qRT-PCR evaluation of COL-10, MMP13, and iNOS expression levels in chondrocytes after 7 days of co-culture with different macrophage CM. (**B**) qRT-PCR evaluation of Aggrecan and Collagen II expression levels in chondrocytes after 7 days of co-culture with different macrophage CM. (**C**) Toluidine blue staining to assess GAG synthesis in chondrocytes after 7 days of co-culture with different macrophage CM. Scale bar = 100 μm. (**D**) Quantitative analysis of sGAG secretion in chondrocytes after 7 days of culture with different macrophage CM. (**E**) CCK-8 assay to evaluate chondrocyte viability at 24 h and 48 h after culture in different macrophage CM. (**F**)–(**G**) Quantitative analysis of chondrocyte apoptosis rate using flow cytometry and V-FITC/PI apoptosis assay after culture in different macrophage CM. (**H**)–(**I**) Quantitative analysis of apoptosis-related protein expression levels (BAX, BCL-2, Caspase-3, and cleaved Caspase-3) using Western blotting. N = 3. *Significant difference compared to the M1-CM group: ***p < 0.001. Abbreviations: BIA: Biocompatible ionized air; CM: conditioned media; DMEM: Dulbecco’s modified eagle medium; COL-10: Collagen-10; MMP13: Matrix metalloproteinase 13; iNOS: inducible nitric oxide synthase; GAG: Glycosaminoglycan; BAX: BCL-2-associated X protein; BCL-2: B-cell lymphoma 2.

Chondrocyte viability assessment (Fig. 5E) showed that, compared to the DMEM, M0-CM, and M2-CM groups, chondrocyte viability was notably reduced in the M1-CM group. In contrast, chondrocyte viability in the M1^BIA180^-CM group, indirectly affected by BIA, significantly increased compared to the M1-CM group. These findings suggest that by promoting M1-to-M2 macrophage polarization, BIA can alleviate the negative impact on chondrocyte viability.

Apoptosis rate analysis (Fig. 5F and G) revealed that chondrocyte apoptosis was significantly elevated in the M1-CM group compared to the DMEM, M0-CM, and M2-CM groups. In contrast, the chondrocyte apoptosis rate in the M1^BIA180^-CM group, indirectly influenced by BIA, markedly decreased compared to the M1-CM group. Apoptosis-related protein analysis (Fig. 5H and I) indicated that, compared to the DMEM, M0-CM, and M2-CM groups, the expression of the anti-apoptotic factor Bcl-2 was reduced in the M1-CM group. In contrast, pro-apoptotic factors BAX and cleaved Caspase-3 were elevated. However, in the M1^BIA180^-CM group, Bcl-2 expression increased, and BAX and cleaved Caspase-3 levels decreased compared to the M1-CM group.These results suggest that M1 macrophages promote chondrocyte apoptosis, while BIA can inhibit M1 macrophage-induced chondrocyte apoptosis by promoting M1-to-M2 polarization.

### BIA alleviates OA rat articular cartilage damage, reduces osteophyte formation, and bone resorption

**Fig. 6:**
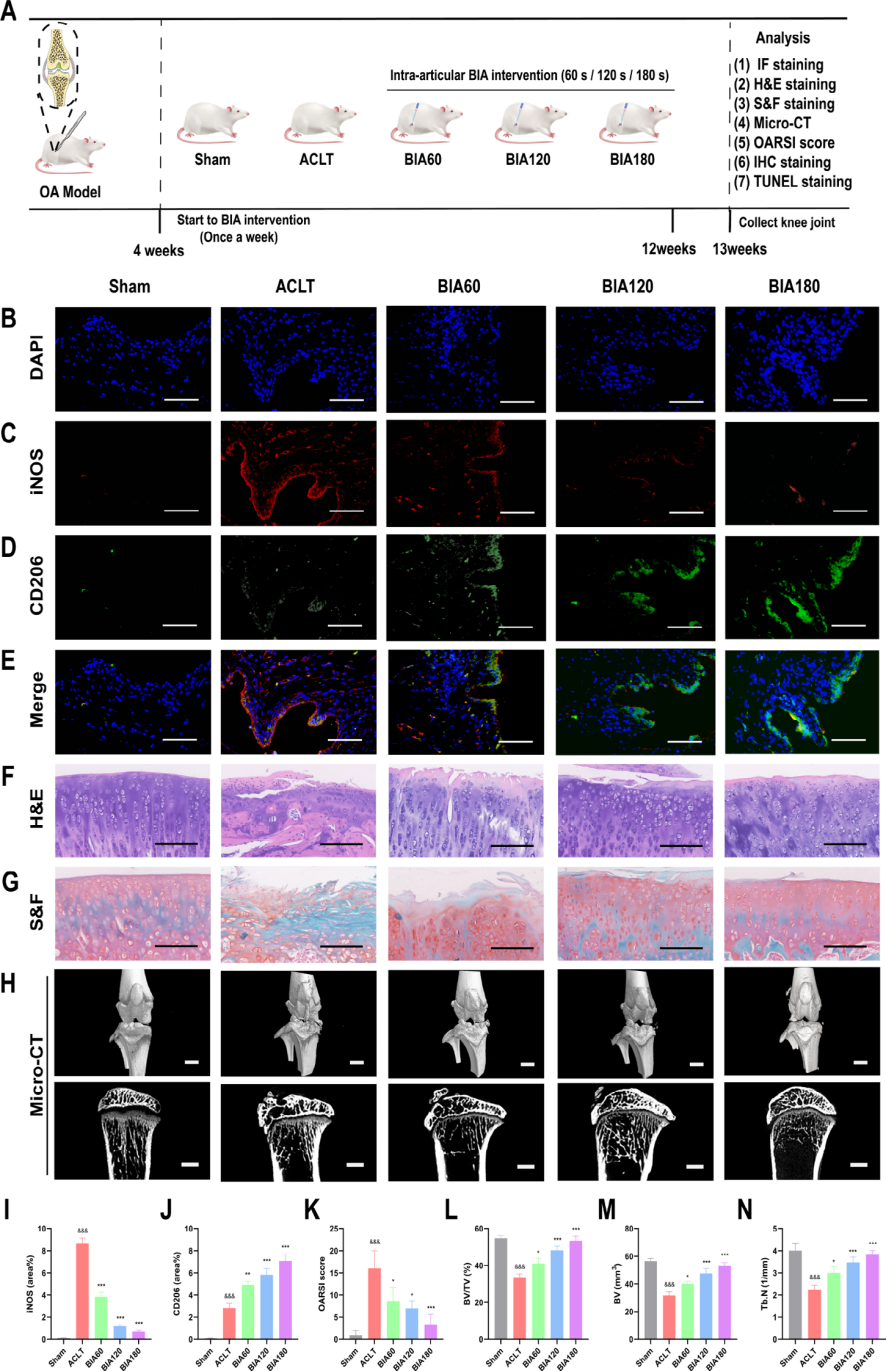
In vivo effect of BIA treatment on rat knee OA. (**A**) Experimental design and schematic illustration of the rat knee OA model and evaluation of BIA’s protective effects. (**B**)–(**E**) Immunofluorescent staining of M1 (iNOS-positive) and M2 (CD206-positive) macrophages in the synovium. DAPI stains cell nuclei. Scale bar = 20 μm. (**F**) Hematoxylin and Eosin (H&E) staining. Scale bar = 50 μm. (**G**) Safranin and Fast Green (S&F) staining. Scale bar = 50 μm. (**H**) Three-dimensional micro-CT images (scale bar = 5 mm) and sagittal views of the knee joint (scale bar = 2 mm). (**I**)–(**N**) Quantitative analysis of M1/M2 macrophages, OARSI scores, BV, BV/TV, and Tb.N. N = 3. *Significant difference compared to the ACLT group: *p < 0.05, **p < 0.01, ***p < 0.001. ^&^Significant difference compared to the Sham group: ^&&&^p < 0.001. Abbreviations: BIA: Biocompatible ionized air; OA: Osteoarthritis; iNOS: inducible nitric oxide synthase; CD206: Cluster of Differentiation 206; ACLT: Anterior cruciate ligament transection; DAPI: 4',6-diamidino-2-phenylindole; H&E: Hematoxylin and eosin Staining; S&F: Safranin fast green staining; OARSI: Osteoarthritis research society international; BV: Bone volume; TV: Tissue volume; Tb.N: Trabecular number.

An anterior cruciate ligament transection (ACLT) and medial meniscus removal established a rat knee OA model. Four weeks post-surgery, rats received intra-articular BIA treatment for 8 weeks. Knee joints were then collected for immunofluorescence, histological staining, Micro-CT, and 3D reconstruction (Fig. 6A). IF staining (Fig. 6B, C, D, E, I, J) demonstrated that, compared to the Sham group, ACLT rats had a significant increase in M1 macrophages (iNOS-positive cells) in the synovium. In contrast, BIA-treated rats showed a significant decrease in M1 macrophages and an increase in M2 macrophages (CD206-positive cells), indicating that BIA promotes M1-to-M2 macrophage polarization. BIA also showed time-dependent modulation of M1-to-M2 macrophage polarization, consistent with in vitro results. H&E staining revealed cartilage surface discontinuity, reduced thickness, and disrupted tidemark in the ACLT group (Fig. 6F), while S&F staining showed lighter cartilage staining, indicating decreased proteoglycan content (Fig. 6G). In contrast, BIA-treated groups showed improved cartilage integrity and increased proteoglycan content.

### BIA-mediated modulation of M1 to M2 macrophage polarization exerts protective and anti-apoptotic effects on chondrocytes

For 7 days, rat articular chondrocytes were co-cultured with DMEM, M0-CM, M1-CM, M1^BIA180^-CM, and M2-CM, followed by qRT-PCR analysis. The results (Fig. 5A and B) showed elevated COL-10, MMP13, and iNOS expression in chondrocytes in the M1-CM group compared to the DMEM, M0-CM, and M2-CM groups, while Aggrecan and Collagen II expression was reduced. Compared to the M1-CM group, the M1^BIA180^-CM group exhibited reduced COL-10, MMP13, and iNOS expression, alongside increased Aggrecan and Collagen II expression in chondrocytes (Fig. 5A and B). These results indicate that M1 macrophages promote catabolic factors associated with matrix degradation in chondrocytes, significantly inhibiting matrix protein expression. In the BIA intervention group, catabolic factor expression was reduced, and matrix protein expression inhibition was alleviated. GAG levels in chondrocytes were qualitatively and quantitatively analyzed. The results (Fig. 5C and D) showed significantly reduced GAG expression and secretion in the M1-CM group compared to the DMEM, M0-CM, and M2-CM groups. However, the M1^BIA180^-CM group exhibited a substantial increase in GAG expression and secretion compared to the M1-CM group (Fig. 5C and D). These findings indicate that M1 macrophages inhibit GAG expression in chondrocytes, while GAG levels increased following BIA intervention.

### BIA-mediated modulation of M1 to M2 macrophage polarization exerts protective and anti-apoptotic effects on chondrocytes

For 7 days, rat articular chondrocytes were co-cultured with DMEM, M0-CM, M1-CM, M1^BIA180^-CM, and M2-CM, followed by qRT-PCR analysis. The results (Fig. 5A and B) showed elevated COL-10, MMP13, and iNOS expression in chondrocytes in the M1-CM group compared to the DMEM, M0-CM, and M2-CM groups, while Aggrecan and Collagen II expression was reduced. Compared to the M1-CM group, the M1^BIA180^-CM group exhibited reduced COL-10, MMP13, and iNOS expression, alongside increased Aggrecan and Collagen II expression in chondrocytes (Fig. 5A and B). These results indicate that M1 macrophages promote catabolic factors associated with matrix degradation in chondrocytes, significantly inhibiting matrix protein expression. In the BIA intervention group, catabolic factor expression was reduced, and matrix protein expression inhibition was alleviated. GAG levels in chondrocytes were qualitatively and quantitatively analyzed. The results (Fig. 5C and D) showed significantly reduced GAG expression and secretion in the M1-CM group compared to the DMEM, M0-CM, and M2-CM groups. However, the M1^BIA180^-CM group exhibited a substantial increase in GAG expression and secretion compared to the M1-CM group (Fig. 5C and D). These findings indicate that M1 macrophages inhibit GAG expression in chondrocytes, while GAG levels increased following BIA intervention.

Furthermore, the OARSI scores were lower in each BIA-treated group than in the ACLT group, indicating that BIA alleviated OA-related cartilage degeneration (Fig. 6K). Micro-CT 3D reconstruction (Fig. 6H) of knee joints showed a significant increase in osteophyte formation in the ACLT group compared to the Sham group. However, BIA-treated groups showed a notable reduction in osteophyte formation. Sagittal CT images of subchondral bone beneath the tibial plateau are demonstrated in Fig. 6H. Quantitative analysis revealed significant decreases in subchondral bone BV, BV/TV, and Tb.N in the ACLT group compared to the Sham group (Fig. 6L, M, N). In contrast, BIA-treated groups showed a time-dependent increase in BV, BV/TV, and Tb.N compared to the ACLT group (Fig. 6L, M, N). These findings suggest that OA increases subchondral bone loss, while BIA time-dependently reduces bone loss and inhibits subchondral bone remodeling.

### BIA alleviates cartilage damage and delays the progression of OA

**Fig. 7:**
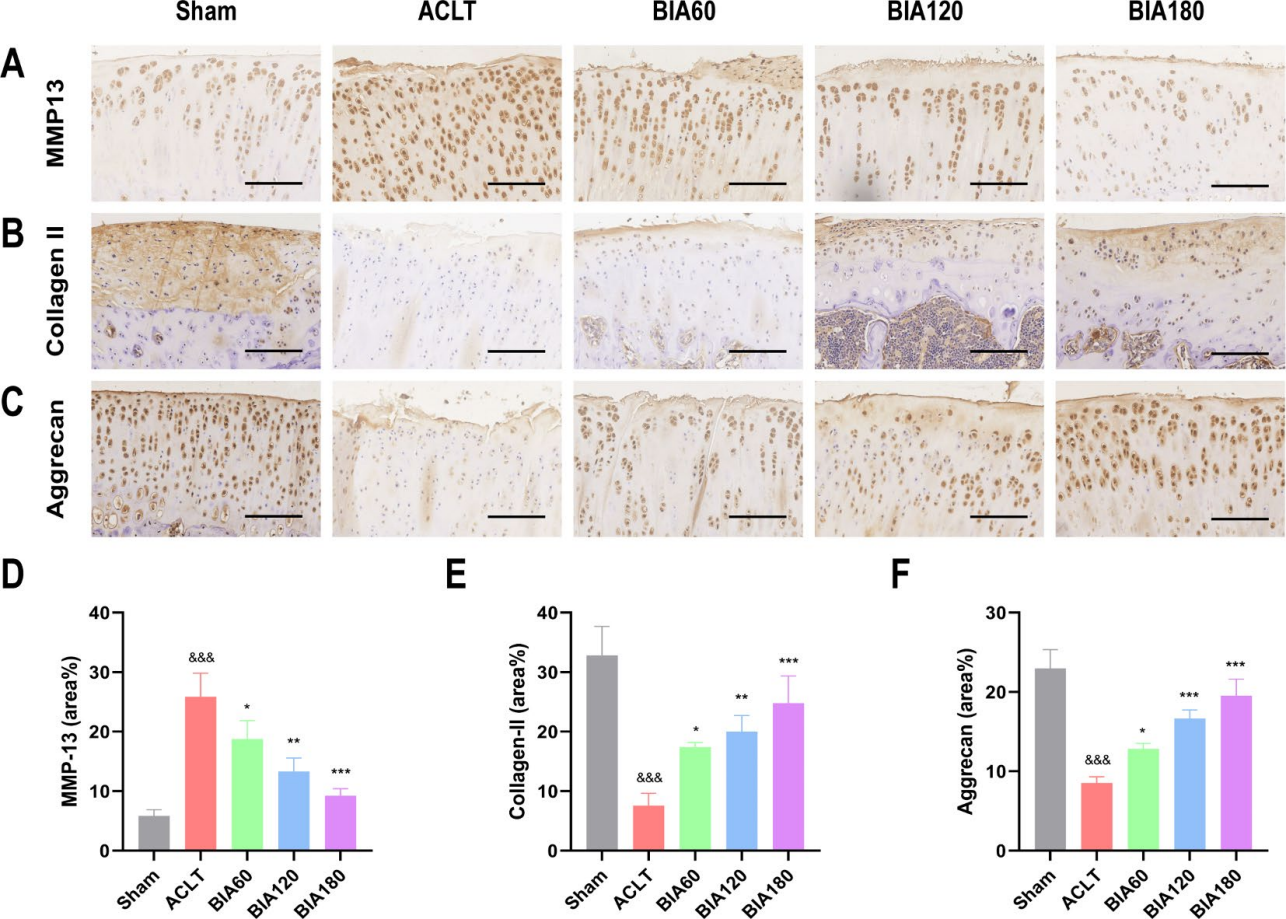
Further staining evaluation of BIA treatment on rat OA. (**A**) Immunohistochemical staining of MMP-13. Scale bar = 50 μm. (**B**) Immunohistochemical staining of Collagen II. Scale bar = 50 μm. (**C**) Immunohistochemical staining of Aggrecan. Scale bar = 50 μm. (**D**)–(**F**) Quantitative analysis of MMP-13, Collagen II, and Aggrecan. N = 3. *Significant difference compared to the ACLT group: *p < 0.05, **p < 0.01, ***p < 0.001. ^&^Significant difference compared to the Sham group: ^&&&^p < 0.001. Abbreviations: BIA: Biocompatible ionized air; OA: Osteoarthritis; ACLT: Anterior cruciate ligament transection; TUNEL: Terminal deoxynucleotidyl transferase dUTP; MMP-13: Matrix metalloproteinase 13.

IHC staining (Fig. 7A–F) showed that MMP-13 expression in the OA group’s articular cartilage significantly increased compared to the Sham group, while Collagen II and Aggrecan deposition significantly decreased. Encouragingly, BIA treatment resulted in a time-dependent decrease in MMP-13 expression and a time-dependent increase in Collagen II and Aggrecan deposition in BIA-treated groups. These results suggest that BIA can time-dependently alleviate cartilage damage and delay OA progression.

### Mechanisms of BIA in alleviating osteoarthritis

**Fig. 8:**
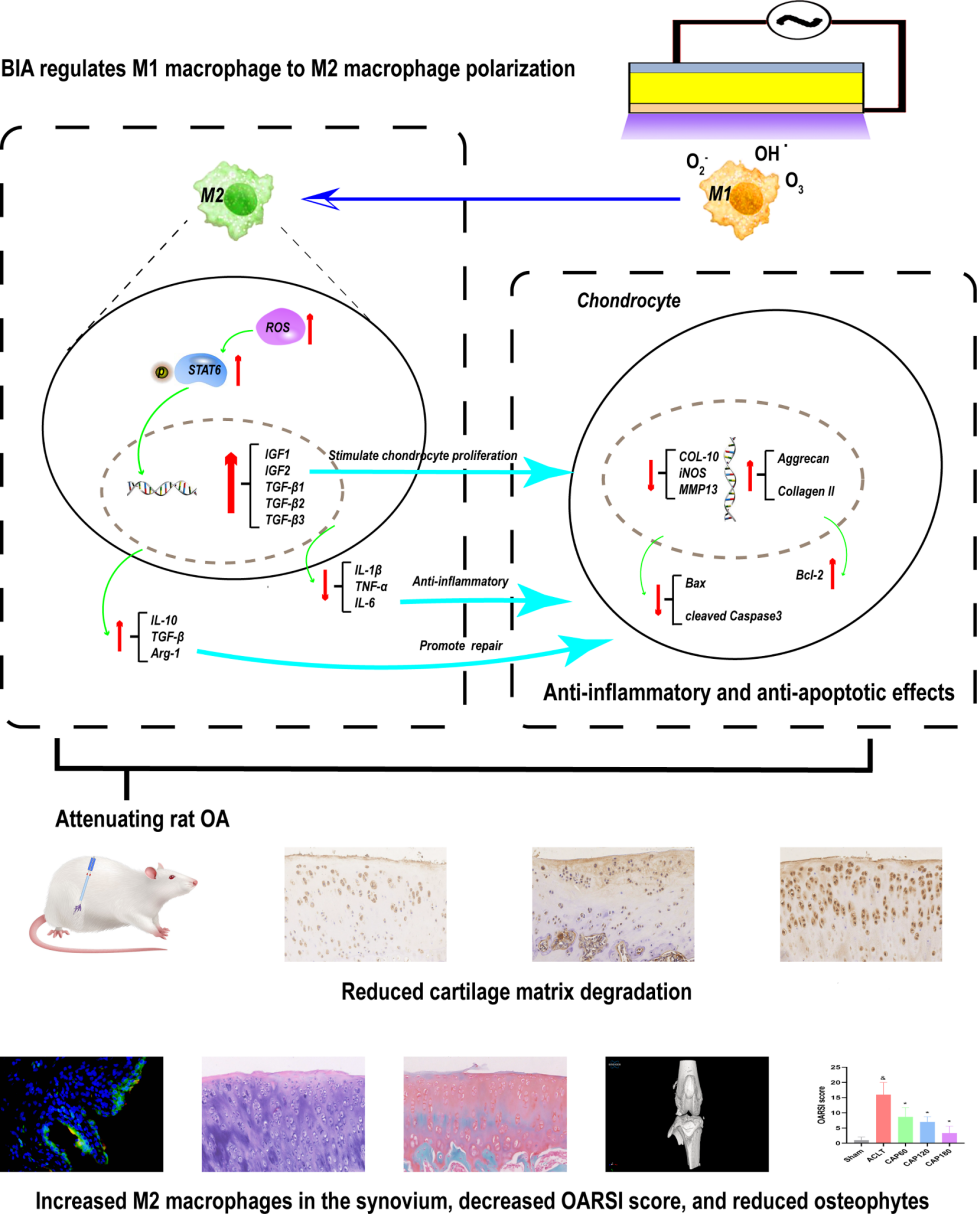
Mechanisms of BIA in Alleviating Osteoarthritis.

Based on the results of in vitro and in vivo experiments, this study outlines how BIA alleviates OA, as illustrated in Fig. 8. The ROS generated by BIA drives the polarization of pro-inflammatory M1 macrophages into anti-inflammatory M2 macrophages, shifting the osteoarthritic microenvironment from pro-inflammatory to anti-inflammatory, thereby reducing cartilage damage. Concurrently, M2 macrophages upregulate anabolic factors involved in cartilage synthesis, fostering a microenvironment conducive to cartilage regeneration and promoting the synthesis of collagen II and proteoglycans by chondrocytes. Additionally, BIA-mediated polarization of M1 macrophages enhances chondrocyte viability, inhibits apoptosis, and decreases cartilage degradation, ultimately leading to the attenuation of OA.

## Discussion

The ability of BIA to regulate macrophage polarization makes it a potential treatment for OA. However, concerns arise about the use of ROS in OA treatment, as previous research has shown the harmful effects of long-term, high-dose ROS on various cells, including chondrocytes^33,34^. Therefore, this study evaluated the impact of low-dose BIA on macrophages and chondrocytes. The results showed that during the 180 s intervention, low-dose BIA had no significant effect on macrophage viability (Fig. 2C), nor did it significantly affect chondrocyte viability or apoptosis (Fig. S1). This suggests that ROS may be a double-edged sword in OA treatment. While prolonged exposure to high concentrations of ROS negatively affects OA, transient increases in low-dose ROS can exert therapeutic effects via various regulatory mechanisms, avoiding harmful outcomes. This aligns with ozone, another ROS, in OA treatment. Ozone therapy has proven safe and effective, promising promising results for pain control and functional recovery in the short to medium term^35,36^. This study, along with previous research, provides theoretical support for the safety of BIA in OA treatment.

This study confirmed that ROS in BIA is the main effector regulating the polarization of M1 to M2 macrophages, with the STAT6 pathway being the primary mechanism of this regulation. This is consistent with the findings of He et al.^32^, whose research also demonstrated that ROS can polarize M1 macrophages into M2 macrophages through the STAT6 pathway. In this study, further analysis revealed an unexpected finding. Even when NAC was used to eliminate ROS from BIA, BIA still induced a lower degree of M1-to-M2 macrophage polarization. This suggests ROS may not be the sole effector in BIA-mediated macrophage polarization. Other effector substances may also regulate M1-to-M2 polarization via the STAT6 pathway. Additionally, although this study ruled out the involvement of common pathways like PI3K-Akt, NF-κB, and Notch, residual M1-to-M2 macrophage polarization was observed after LEF blocked the STAT6 pathway, implying that less common secondary pathways may be involved, warranting further exploration in future studies. Based on these collective results, this study suggests that BIA regulates M1-to-M2 macrophage polarization through ROS via the STAT6 pathway. However, this may only be one of the primary mechanisms. BIA’s regulation of macrophage polarization is likely complex, involving multiple effector molecules acting through various pathways.

The results of this study demonstrate that BIA downregulates the expression of pro-inflammatory mediators IL-1β, TNF-α, and IL-6 in macrophages in a time-dependent manner (Fig. 4A, B, C). Simultaneously, BIA upregulates the expression of anti-inflammatory mediators IL-10, TGF-β, and Arg-1 (Fig. 4D, E, F). BIA terminates the pro-inflammatory cascade by modulating M1 macrophages to the M2 phenotype, resulting in an anti-inflammatory and reparative microenvironment. A previous study indicated that controlling joint inflammation alone is insufficient; improving the local microenvironment for cartilage formation is also necessary^37^. Research suggests that squid type II collagen creates an environment conducive to cartilage formation and repair by promoting macrophages to express pro-chondrogenic factors^16^. This study demonstrates that BIA promotes the expression of pro-chondrogenic factors IGF1, IGF2, TGF-β1, TGF-β2, and TGF-β3 in macrophages (Fig. 4G-P). This suggests that BIA creates a chondrogenic microenvironment by regulating macrophage polarization, potentially aiding cartilage repair.

During the progression of OA, MMP-13 expression increases significantly. MMP-13 degrades cartilage extracellular matrix, such as Collagen II and proteoglycans^38^. COL-10 is widely used to assess chondrocyte status and is a marker for hypertrophic chondrocytes^39,40^. This study found that M1 macrophages enhance the expression of catabolic factors like COL-10, MMP-13, and iNOS in chondrocytes (Fig. 5A), significantly inhibiting matrix protein expression (Aggrecan, Collagen II) (Fig. 5B). However, after BIA-mediated polarization of M1 macrophages to M2 macrophages, catabolic factor expression was reduced (Fig. 5A), and matrix protein expression was restored (Fig. 5B). These effects are mainly attributed to reduced pro-inflammatory mediators following M1-to-M2 polarization.

Increased GAG expression reflects enhanced anabolic activity in cartilage and is widely used as a marker for cartilage synthesis and repair^4,16,41^. This study analyzed GAG expression in chondrocytes across different groups (Fig. 5C and D). The results showed that M1 macrophages inhibited GAG expression in chondrocytes, and increased in the BIA intervention group. This suggests that M1 macrophages suppress chondrocyte anabolic activity, while BIA-induced M1-to-M2 polarization enhances it. The improved anabolic activity is attributed to reduced pro-inflammatory mediators, increased anti-inflammatory mediators, and chondroprotective factors following M1-to-M2 polarization.

Research indicates that M1 macrophages, as pro-inflammatory cells, secrete various pro-inflammatory cytokines, including IL-1β, which can reduce chondrocyte viability^42,43^. This aligns with the findings of this study. Compared to the control groups (DMEM, M0-CM, and M2-CM), the results show that chondrocyte viability significantly decreases in the M1-CM group. Conversely, chondrocyte viability substantially increases in the M1^BIA180^-CM group compared to the M1-CM group (Fig. 5E). The study attributes the improved chondrocyte viability to reduced pro-inflammatory factors and enhanced anti-inflammatory characteristics following BIA-induced M1-to-M2 macrophage polarization. The results of the chondrocyte apoptosis study suggest that BIA can alleviate M1 macrophage-induced apoptosis, potentially helping to slow cartilage degeneration.

As noted in the literature^44^, reducing M1 macrophage infiltration helps alleviate synovial inflammation and slow the progression of OA. Animal experiments show that BIA also exerts a time-dependent effect in vivo, promoting M1 macrophages to adopt the M2 phenotype and significantly reducing M1 macrophage infiltration in the synovium of rats with OA. Histological assessments using H&E and S&F staining further indicate that BIA-mediated macrophage polarization effectively mitigates cartilage damage, improves cartilage structural integrity, and lowers OARSI scores in rats.

A characteristic feature of OA is the degradation and loss of various joint tissues, such as articular cartilage and subchondral bone^45^. The loss of subchondral bone mass is strongly associated with damage to articular cartilage in the early stages of OA^15^. Multivariate analysis was conducted on each rat group’s tibial plateau subchondral bone. The results show that subchondral bone loss accompanies early-stage OA, and the degree of cartilage damage corresponds to the extent of bone loss (Fig. 6H). Importantly, BIA exhibits a time-dependent reduction in bone loss. This study suggests that this is related to the reduced IL-6 expression following the shift of M1 macrophages to the M2 phenotype. As IL-6 is a key cytokine that triggers changes in subchondral bone, promoting osteoclastogenesis, which increases bone resorption and leads to subchondral bone loss^46^. A significant decrease in knee osteophytes was also observed in the OA rat groups that underwent BIA intervention. The literature has documented that osteophyte formation in OA is closely linked to synovial inflammation induced by synovial macrophages^15^. Thus, the study posits that BIA’s modulation of M1-to-M2 macrophage polarization in the synovium, leading to reduced synovial inflammation, is a key factor in the reduction of osteophyte formation in the knee joints of OA rats.

Research indicates that inhibiting MMP-13 production can effectively slow OA progression^47^. IHC results reveal a significant increase in MMP-13 expression in the ACLT group joints. In contrast, BIA-treated groups show varying degrees of reduced MMP-13 expression in the articular cartilage (Fig. 7A and D). Literature indicates that pro-inflammatory mediators like IL-1β stimulate chondrocytes to upregulate MMP-13 expression^48,49^. BIA-induced M1-to-M2 macrophage polarization in the synovium likely reduces pro-inflammatory mediators, including IL-1β. This reduction in pro-inflammatory mediators may decrease MMP-13 expression in chondrocytes, mitigating extracellular matrix degradation. Furthermore, the results indicate reduced deposition of Collagen II and Aggrecan in the ACLT group, while BIA-treated groups show a significant increase in Collagen II and Aggrecan deposition (Fig. 7B, C, E, F). This suggests enhanced cartilage synthesis in BIA-treated rats, promoting cartilage repair in OA.

This study represents the first application of BIA in OA treatment, with promising results. However, several limitations remain. Firstly, as this study focuses on the regulatory effects of BIA on macrophages, only the safety of low-dose BIA on chondrocytes was evaluated. Further research is needed to assess the direct impact of BIA on chondrocytes. Secondly, precise dosing is difficult due to ROS’s high reactivity and short lifespan. While this study indirectly assessed BIA’s ROS content by detecting H_2_O_2_ generated through ROS interactions with liquids, according to the German DIN SPEC 91315 "General Requirements for Medical Plasma Sources"^50^, it provides only a rough estimate of ROS dosage in BIA. This limits the analysis of the dose–response relationship for BIA’s biological effects. Accurate methods for measuring ROS dosage in BIA need further development. Lastly, this study observed that BIA intervention can delay OA progression in animal experiments, likely by preventing cartilage degradation. While in vitro experiments demonstrated BIA’s potential to promote cartilage repair via macrophage polarization, whether BIA intervention in the animal model promotes cartilage regeneration during OA’s delayed progression remains unclear. A more comprehensive experimental design is necessary to address this, including dynamic monitoring and data collection of OA progression at 4 and 8 weeks. The absence of such a design is one of the limitations of this study.

## Conclusion

In vitro experiments demonstrated that BIA promotes M1-to-M2 macrophage polarization through the ROS-mediated STAT6 pathway. This shift reduced pro-inflammatory mediators and increased anti-inflammatory mediators and pro-chondrogenic factors, creating a protective microenvironment for cartilage. In OA rat models, BIA reduced M1 macrophage infiltration and increased the proportion of M2 macrophages in the synovium, inhibited MMP13 production and chondrocyte apoptosis, alleviated cartilage destruction, and delayed OA progression. In conclusion, this study demonstrated the beneficial role of BIA in OA treatment, suggesting its potential as a novel therapeutic option for OA.

## Materials and methods

### Utilization protocols and components of ex vivo and in vivo BIA devices

This study employed micro-arc oxidation and plasma coating processes to fabricate a dielectric barrier discharge (DBD) flat plate measuring 65 mm by 10 mm (Fig. 1A), serving as the ex vivo BIA device (BIA-E). The detailed fabrication process has been described in our previous research^51^. This DBD plate directly ionizes the air to generate BIA. BIA is generated by initiating reactions with the cells beneath it by placing the plate horizontally about 2 cm above the cells and activating the high-voltage AC power supply. The device operates at 5.2 W, with an effective voltage of 1.7 kV and a current of 67 mA.

Figure 1B illustrates the schematic of the in vivo BIA (BIA-I) device for rat joint manipulation. It consists of an adjustable air injection pump, an air conduit, a specially coated insulated metal puncture needle, and a high-voltage electrode. The device operates at 2.1 W with an effective voltage of 700 V and a current of only 3 mA, minimizing the risk of harm to the rats during operation. In the in vivo experiments, the cathode was connected to the rat’s right hind limb at zero potential, and the anode to a metal puncture needle. The injection pump parameters were adjusted to slowly deliver 1 ml of air through the conduit and needle into the rat’s right knee joint. Once the high-voltage AC power source is activated, the voltage difference between the needle tip and surrounding tissue ionizes the air within the joint cavity, generating BIA that interacts with joint tissues, including the synovium. Furthermore, in the in vivo BIA apparatus, the insulation on the metal puncture needle, except for the discharge tip, ensures that all portions of the needle in contact with tissue are insulated, preventing damage and enhancing rats’ safety.

In this study, a fiber optic probe (Ocean Optics 2000, Ocean Optics, USA) was used for spectral analysis of the BIA generated by both in vitro and in vivo devices. The probe captures spectra within 300–1000 nm range, enabling BIA composition analysis. ROS levels generated by the BIA-E and BIA-I devices were indirectly assessed using an H_2_O_2_ assay kit (BC3595, Solarbio, China). This method is based on forming of a yellow peroxide-titanium complex through the reaction of H_2_O_2_ with titanium sulfate, which absorbs at 415 nm.

To measure H_2_O_2_, 2 ml of Dulbecco’s Modified Eagle’s Medium (DMEM; G4510, Servicebio, China) was treated with the BIA-E and BIA-I devices for different durations (0 s, 60 s, 120 s, and 180 s). Then, following the H_2_O_2_ detection kit guidelines, 0.1 ml of treated DMEM was extracted for testing. An infrared thermal imager (FLIR ONE, USA) was used to measure the operational temperatures of both BIA devices. The BIA-E and BIA-I devices were placed on a cool-toned background panel, centered in the camera’s field of view, with the ambient temperature maintained between 20 °C and 25 °C.

### Cell isolation, culture, treatment and preparation of conditioned medium

The cell experiment protocol was approved by the Ethics Committee of Anhui Medical University (No.: LISC20231141). RAW264.7 cells (M0 macrophages) were obtained from the Stem Cell Bank of the Chinese Academy of Sciences, Shanghai, China. M0 macrophages were seeded in a 6-well plate at 2 × 10^5^ cells/mL and cultured in DMEM supplemented with 10% fetal bovine serum and 1% penicillin-streptomycin at 37 °C in a humidified incubator with 5% CO_2_. Once optimal growth was achieved, M0 macrophages were induced to polarize to the M2 phenotype by adding 20 ng/mL IL-4 (P6267, Beyotime Biotechnology, China) for 24 h. M1 macrophages were induced by adding 20 ng/mL IFN-γ (P6398, Beyotime Biotechnology, China) and 100 ng/mL LPS (ST1470, Beyotime Biotechnology, China) for 24 hours^52^.

To investigate whether BIA regulates M1 macrophage polarization to M2 through the ROS-mediated STAT6 pathway, this study used Leflunomide (LEF; HY-B0083, MCE, USA) and N-acetyl-L-cysteine (NAC; HY-B0215, MCE, USA) to pretreat M1 macrophages. LEF, a STAT6 inhibitor, blocks the STAT6-dependent pathway by preventing STAT6 phosphorylation. NAC, a potent ROS scavenger, eliminates ROS in BIA-treated M1 macrophages. M1 macrophages were pretreated with 75 μM LEF for 12 h to block STAT6, or with 20 mM NAC for 1 h to enhance ROS scavenging, followed by BIA intervention. This approach enables the study of how BIA regulates macrophage polarization.

The method for isolating and culturing chondrocytes was consistent with established techniques used in our previous study^53^. Chondrocytes from passages 1 to 3 were used for subsequent experiments. To compare the impact of BIA-treated macrophages on chondrocytes, this study collected supernatants from macrophages subjected to different conditions as conditioned media (CM) for culturing chondrocytes. This approach enables the investigation of macrophage-secreted factors on chondrocyte behavior under varying conditions. M0 macrophages, M1 macrophages, BIA-treated M1 macrophages (treated for 180 s), and M2 macrophages were cultured in serum-free medium (11,965,092, Thermo Fisher, China) at 37 °C in a CO_2_ incubator for 24 h. Supernatants from each macrophage group were collected by centrifugation (1200 rpm, 5 min) to remove cells, then diluted 1:1 with serum-free DMEM to prepare CM for chondrocytes, including M0-CM, M1-CM, M1^BIA180^-CM, and M2-CM. The obtained CM was used for subsequent chondrocyte experiments.

### Cell viability assay

Cell viability of M1 macrophages and chondrocytes was assessed using the CCK-8 assay kit (C0037, Beyotime Biotechnology, China). For M1 macrophages, 3 × 10^3^ cells per well were seeded into a 96-well plate. After BIA treatment for 0 s, 60 s, 120 s, or 180 s, cells were cultured for 24 h. Then, 10 µL of CCK-8 reagent was added to each well and incubated at 37 °C in 5% CO_2_ for 2 h. Absorbance at 450 nm was measured using a microplate reader (Bio-Rad, Hercules, CA, USA). 5 × 10^3^ cells per well were seeded in a 96-well plate for chondrocytes were seeded in a 96-well plate and cultured for 24 h. Then, different media (DMEM, M0-CM, M1-CM, M1^BIA180^-CM, and M2-CM) were used for 24-h and 48-h incubations, followed by CCK viability assay.

### Intracellular ROS contents

The ROS Assay Kit (S0033S, Beyotime Biotechnology, China) was used to assess changes in intracellular ROS levels in macrophages before and after BIA treatment. After BIA treatment for 0 s, 60 s, 120 s, or 180 s, cells were incubated for 24 h at 37 °C in 5% CO_2_. ROS detection was performed according to the manufacturer’s instructions.

### Cytokines measurement

The enzyme-linked immunosorbent assay (ELISA) was used to assess the expression of multiple pro-inflammatory and anti-inflammatory cytokines in M1 macrophages after BIA treatment. M1 macrophages were treated with BIA for 0 s, 60 s, 120 s, or 180 s, then cultured for 24 h. Supernatants were collected from the cultures. Following the instructions of each ELISA kit (Meimian, China), the levels of IL-1β, IL-6, TNF-α, IL-10, TGF-β, Arg-1, IGF1, IGF2, TGF-β1, TGF-β2, and TGF-β3 were measured.

### Immunofluorescence (IF) staining

M1 macrophages were treated with BIA for varying durations (0 s, 60 s, 120 s, 180 s) and cultured for 24 h at 37 °C in a 5% CO2 incubator. The treated cells were fixed in 0.1% Triton X-100 paraformaldehyde. Cells were blocked with 4% goat serum to prevent nonspecific binding. Subsequently, cells were incubated overnight with primary antibodies against iNOS (1:200; AF0199, Affinity, China), CD206 (1:200; 60,143–1-lg, Proteintech, China), or p-STAT6 (1:400; AF3301, Affinity, China). To ensure binding specificity and exclude nonspecific staining, isotype control antibodies matching the source and IgG subtype of the primary antibodies were used in parallel: for rabbit-derived primary antibodies (anti-iNOS and p-STAT6), Rabbit IgG monoclonal [EPR25A]—Isotype Control (1:200; ab172730, Abcam, UK) was used, and for mouse-derived primary antibodies (anti-CD206), Mouse IgG2a kappa Isotype Control (1:200; HY-P99978, MedChemExpress, USA) was used. The cells were stained with fluorescent secondary antibodies and 4′,6-diamidino-2-phenylindole (DAPI; G1012, Servicebio, China). Isotype control samples were processed identically to experimental samples. The cells were observed under a fluorescence microscope (LSM8800, Carl Zeiss, China). iNOS-positive cells were identified as M1 macrophages, and CD206-positive cells as M2 macrophages.

### Quantitative reverse transcription polymerase chain reaction analysis (qRT-PCR)

qRT-PCR was used to assess the polarization shift of M1 macrophages, chondrogenic, and chondrocyte function-related genes after BIA intervention. Total RNA from macrophages and chondrocytes was extracted using TRIzol reagent (RP1001, BioTeke, China). RNA was reverse-transcribed into cDNA using the Prime Script RT Master Mix kit (6210A, Takara, Japan). PCR was performed to obtain Ct values using the TB Green PreMix Ex Taq kit (RR42LR, Takara, Japan). Values were normalized to β-actin mRNA levels, and gene expression was calculated using the 2^-ΔΔCt^ method. The primer sequences are listed in Table S1.

### Toluidine blue staining

To observe glycosaminoglycan (GAG) secretion from chondrocytes, the cells were cultured in dishes with the corresponding conditioned medium (CM) for 7 days. They were then fixed with 4% paraformaldehyde and washed twice with PBS. Chondrocytes were stained with toluidine blue (G2543, Solarbio, China), and stained GAG in the chondrocytes was observed under an optical microscope (DM2000, Leica, Germany).

### Sulfated glycosaminoglycan quantification

Chondrocytes were seeded at 2 × 10^5^ cells/well in a 6-well plate and cultured for 7 days using the corresponding macrophage CM. After 7 days, before collecting the supernatant for sGAG detection, cell viability in each group was assessed using the Trypan Blue exclusion method. The cell suspension was mixed with 0.4% Trypan Blue solution (PB180423, Procell, Wuhan) at a 1:1 ratio. The stained cell suspension was loaded onto a Neubauer hemocytometer, and the number of viable cells was counted under a light microscope. sGAG concentrations in the supernatant were measured following the manufacturer’s instructions using the Blyscan™ sGAG assay kit (B1000, BioVendor, Czechia). The sGAG concentration per 100,000 viable cells (µg/10^5^ cells) was calculated to ensure data comparability.

### Flow cytometric analysis

To detect the apoptosis rate of chondrocytes cultured in different CM, cells were seeded at 5 × 10^5^ cells per well in 6-well plates and cultured with DMEM, M0-CM, M1-CM, M1^BIA180^-CM, or M2-CM for 7 days. After centrifugation (150 rpm, 5 min), cells were washed, counted, and collected. Following the manufacturer’s instructions for the Cell Apoptosis Detection Kit (C1062M, Beyotime Biotechnology, China), cells were resuspended in 500 μl of 1X Annexin V Binding Buffer. Then, 5 μl Annexin V-FITC and 10 μl Propidium Iodide were added and mixed, followed by incubation in the dark for 15 min. Flow cytometry assessed chondrocyte apoptosis (NovoCyte, Agilent, USA).

### Western blot analysis

After treatments, macrophages and chondrocytes were washed three times with PBS and lysed using pre-cooled RIPA lysis buffer (P0013B, Beyotime Biotechnology, China). The supernatant was collected following centrifugation, and protein concentration was assessed using the BCA protein assay kit (P0012S, Beyotime Biotechnology, China). In each group, 40 μg of protein was separated using 8–12% SDS-PAGE and transferred onto a polyvinylidene fluoride (PVDF) membrane. After 60 min of blocking with milk, the PVDF membrane was incubated with primary antibodies overnight at 4 °C. The membrane was washed thrice with Tris-buffered saline with Tween 20 (TBST) and incubated with specific secondary antibodies at 37 °C for 1 h. The membrane was washed three times with TBST and incubated with chemiluminescent horseradish peroxidase (HRP) substrate (A0208, Beyotime Biotechnology, China). Images were visualized using the Tanon 5200 system (Tanon, China) and analyzed with ImageJ (NIH, USA). The primary antibodies used in this study are as follows: iNOS (1:1000; AF0199, Affinity, China), CD206 (1:2000; 60,143–1-lg, Proteintech, China), β-actin (1:1000; AF7018, Affinity, China), STAT6 (1:1000; AF6302, Affinity, China), p-STAT6 (1:1000; AF3301, Affinity, China), AKT (1:1000; AF6261, Affinity, China), p-AKT (1:1000; AF0016, Affinity, China), NF-κB p65 (1:1000; AF5006, Affinity, China), p-NF-κB p65 (1:1000; AF3387, Affinity, China), Cleaved-Notch 1 (1:1000; AF5307, Affinity, China), Notch 1 (1:1000; ab52627, abcam, UK), BAX (1:500; AF0120, Affinity, China), Bcl-2 (1:500; BF9103, Affinity, China), caspase-3 (1:500; WL0003b, Wanleibio, China).

### Animal experiments

Sprague–Dawley (SD) rats (8 weeks old, male, SPF grade, 280-320 g) were purchased from Liaoning Changsheng Biotechnology Technology Company Limited (Certificate No: 210726221101541372). After a week of acclimatization, thirty SD rats were randomly divided into five groups, each with six rats: Sham group, OA group, BIA60 group, BIA120 group, and BIA180 group. The anterior cruciate ligament transection and medial meniscus resection established the OA model. After anesthesia was induced in rats via intraperitoneal injection of 3% pentobarbital sodium (30 mg/kg, p3761, Sigma, USA), the right knee was shaved and disinfected, and a medial parapatellar incision was made to expose the joint. The anterior cruciate ligament was transected, and the medial meniscus was removed. The joint capsule and skin were sutured. In the Sham group, the joint capsule was only opened for exposure without ligament or meniscus removal.

Four weeks after surgery, the BIA60, BIA120, and BIA180 groups received weekly intra-articular BIA interventions for 8 weeks, with intervention durations of 60 s, 120 s, and 180 s, respectively. The Sham and OA groups received no intervention. One week after the final BIA intervention, all rats were euthanized via intraperitoneal injection of an overdose of pentobarbital sodium. Knee joint specimens were then collected for subsequent experiments. The entire SD rat experimental protocol was approved by the Animal Ethics Committee of Anhui Medical University (Ethics No.: LISC20231141). All experimental methods and procedures involving animals adhered to the guidelines and regulations of the Animal Management Committee of Anhui Medical University and complied with the ARRIVE guidelines.

### Histopathological staining

The right knee joints were fixed in 4% paraformaldehyde (G1101, Servicebio, China) for 48 h. The joints were decalcified in 10% ethylenediaminetetraacetic acid (EDTA; ST066, Beyotime Biotechnology, China) for 8 weeks. After dehydration and paraffin embedding, 5 μm thick sagittal sections were obtained from the medial compartment of the knee joint. The sections were stained with Hematoxylin–Eosin (H&E) and Safranin-O/Fast Green (S&F). OA severity was evaluated using the Osteoarthritis Research Society International (OARSI) scoring system, with higher scores indicating more severe cartilage damage.

### Immunohistochemical (IHC) and IF staining

For IHC staining, after pre-treatments including deparaffinization, clearing, rehydration, blocking, and antigen retrieval, sections were incubated with primary antibodies for 1 h: MMP-13 (1:200; AF5355, Affinity, China), Collagen II (1:200; AF0135, Affinity, China), or Aggrecan (1:200; DF7561, Affinity, China). To ensure antibody specificity and avoid non-specific staining, isotype control antibodies matching the source and IgG subtype of the primary antibodies were used in parallel. For rabbit-derived primary antibodies (e.g., anti-MMP13, anti-Collagen II, anti-Aggrecan), Rabbit IgG, monoclonal [EPR25A]—Isotype Control (1:200; ab172730, Abcam, UK) was used. Sections were then incubated with horseradish peroxidase (HRP)-conjugated secondary antibodies for 30 min at room temperature, developed using diaminobenzidine (DAB; G1212, Servicebio, China), and counterstained with hematoxylin. The isotype control samples were treated identically to the experimental samples.

For IF staining, sections were pre-treated with deparaffinization, clearing, rehydration, blocking, and antigen retrieval. They were then incubated overnight at 4 °C with primary antibodies against iNOS (1:200; Affinity, China) or CD206 (1:200; Proteintech, China), along with their respective isotype control antibodies (1:200; same source and IgG subtype as the primary antibodies). For rabbit-derived primary antibodies (e.g., anti-iNOS), Rabbit IgG, monoclonal [EPR25A]—Isotype Control (1:200; ab172730, Abcam, UK) was used. For mouse-derived primary antibodies (e.g., anti-CD206), Mouse IgG2a kappa, Isotype Control (1:200; HY-P99978, MedChemExpress, USA) was used. Sections were then incubated with fluorescently labeled secondary antibodies for 1 h at room temperature, followed by DAPI (G1012, Servicebio, China) counterstaining for nuclei. The isotype control samples were treated identically to the experimental samples.

Sections were observed under a fluorescence microscope (LSM8800, Carl Zeiss, Shanghai), and three randomly selected high-power fields per section were used to record staining intensity and distribution. Quantitative evaluation and statistical analysis were performed using ImageJ (NIH, USA).

### Micro-CT scanning and index analysis

The right knee joints were collected and fixed in 4% paraformaldehyde (G1101, Servicebio, China). Continuous cross-sectional images of the right knee joint were acquired using Micro-CT (Skyscan1276, Bruker, Belgium) at a resolution of 10 μm. The current and voltage were set to 200 μA and 85 kV, respectively. CTVOX software (IPR, China) was used for 3D reconstruction to observe bone spur formation in the knee joint. Quantitative analysis was performed on the subchondral bone of the tibial plateau, as the Region of Interest (ROI). The 3D structural parameters analyzed included Bone Volume (BV), Bone Volume/Total Volume (BV/TV), and Trabecular Number (Tb.N).

### Statistical analysis

All quantitative data are presented as mean ± standard deviation (SD). Statistical analysis was conducted using Prism 8.3.0 (GraphPad Software Inc., La Jolla, CA, USA). Unpaired two-tailed Student’s t-test or one-way ANOVA was used to assess significant differences between two or more groups. *p* < 0.05 was considered statistically significant.

## Electronic Supplementary Material

Below is the link to the electronic supplementary material.

Supplementary Material 1

## Data Availability

The data supporting this study’s findings are available from the corresponding author upon reasonable request.
