# Supplementary material for: Biocompatible ionized air alleviates rat osteoarthritis by modulating polarization from M1 to M2 macrophages
Source: Sci Rep. 2024 Dec 30;14:31901. doi: 10.1038/s41598-024-83198-6 (PMC11685818; doi:10.1038/s41598-024-83198-6)

Supplementary information

Supplementary Figure 1

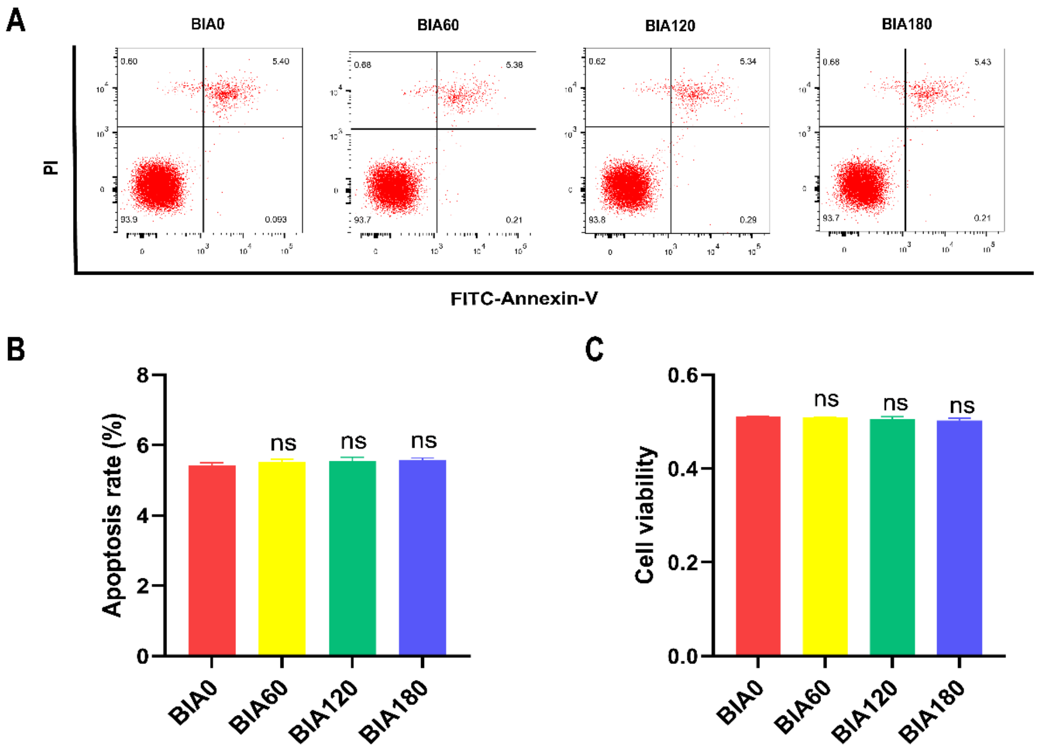

Fig. S1. The impact of BIA on chondrocyte apoptosis and cell viability. (A) Flow cytometry analysis of the effect of different BIA intervention durations on chondrocyte apoptosis. **(B)** Quantitative analysis of the effect of BIA on chondrocyte apoptosis. **(C)** CCK-8 assay to evaluate the effect of different BIA intervention durations on chondrocyte viability. N = 3. 'ns' indicates no significant difference compared to the BIA0 group.

Supplementary Figure 2

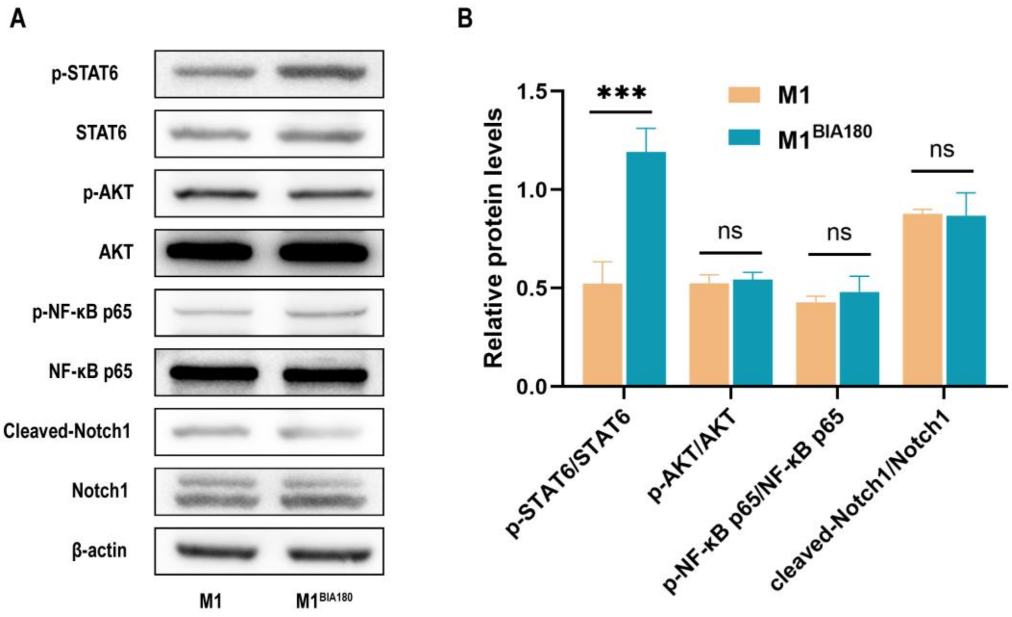

Fig. S2. Activation of key proteins in multiple pathways during BIA-regulated polarization of M1 to M2 macrophages. (A) Western blot analysis of p-STAT6, STAT6, p-AKT, AKT, p-NF-κB p65, NF-κB p65, Cleaved-Notch1, and Notch1 expression levels in macrophages from different groups after BIA intervention. **(B)** Quantitative analysis of p-STAT6, STAT6, p-AKT, AKT, p-NF-κB p65, NF-κB p65, Cleaved-Notch1, and Notch1 expression levels in macrophages. N = 3. *Significant difference between the two groups: ***$p < 0.001$. 'ns' indicates no significant difference between the two groups.

Supplementary Table 1

Tab. S1 Primer sequences used in qRT-PCR.

Target Genes	Forward Primer(5′—3′)	Reverse Primer(5′—3′)
**iNOS**	CACCACCCTCCTCGTTC	CAATCCACAACTCGCTCC
**CD206**	GCAAGTGATTTGGAGGCT	ATAGGAAACGGGAGAACC
**IGF1**	GGTGGATGCTCTTCAGTT	TTTGTAGGCTTCAGTGGG
**IGF2**	TTGTTGACACGCTTCAGTTT	GCAGCACTCTTCCACGAT
**TGF-β1**	GGCGGTGCTCGCTTTGTA	TTTCTCATAGATGGCGTTGTT
**TGF-β2**	CGGAGCGACGAGGAGTA	GGACGATTCTGAAGTAGGGT
**TGF-β3**	CCAGGGCAGTCAGAGGA	TGGGTTCAGGGTGTTGTAT
**Aggrecan**	AAACCTGGCGTGAGAAC	TGTCAGAGGGTGATGTGG
**MMP13**	AGAATCTATGATGGCACTG	TGTAGCCTTTGGAACTG
**Collagen-II**	GGACACCGAGGTTTCAC	CCAGGGATTCCATTAGAG
**COL-10**	GGATGCCGCTTGTCAGT	AGGCGTGCCGTTCTTAT
**β-actin**	CTGTGCCCATCTACGAGGGCTAT	TTTGATGTCACGCACGATTTCC

Original blots/gels of Figure 2 in the manuscript

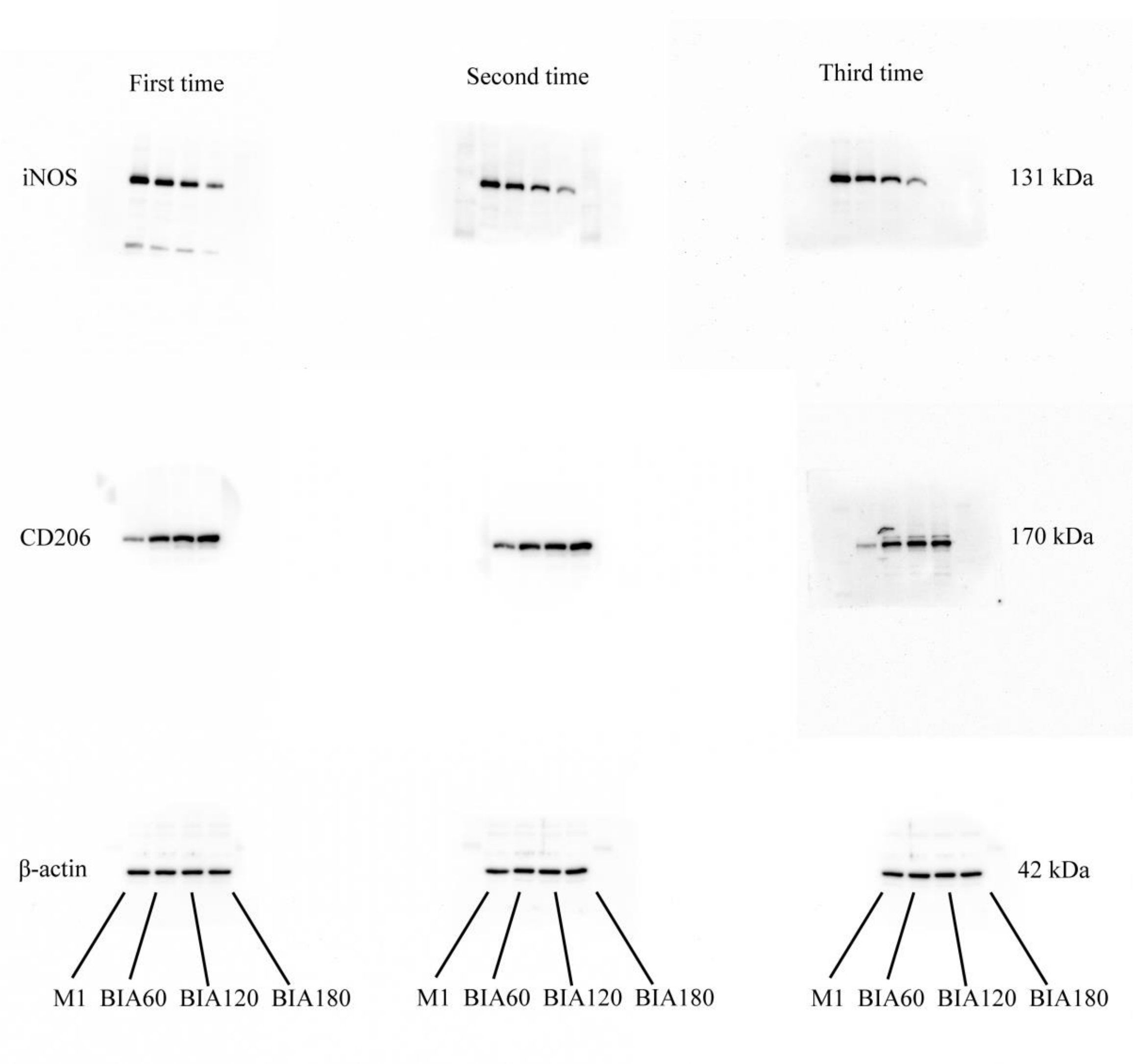

Original blots/gels of Figure 3 in the manuscript

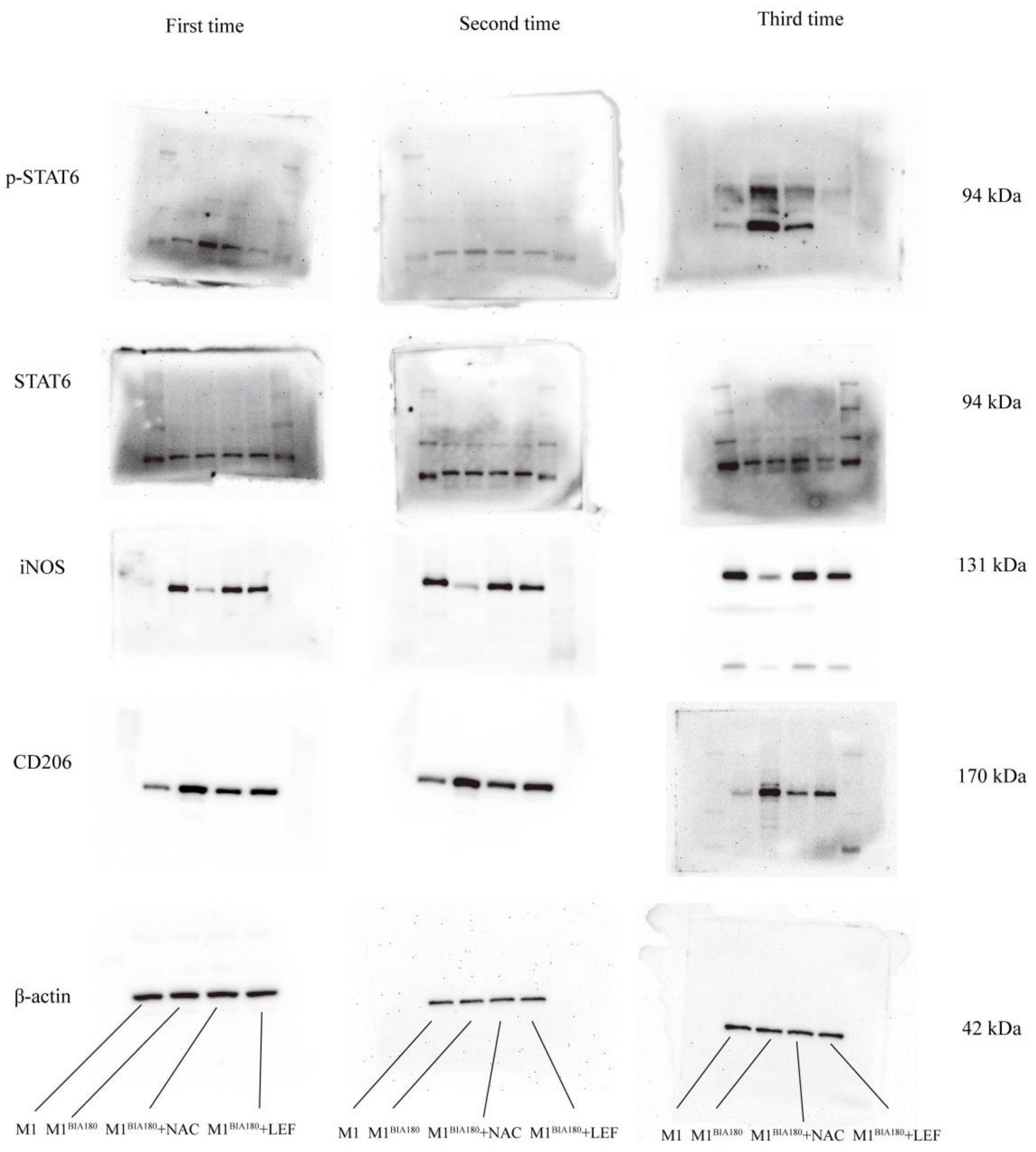

Original blots/gels of Figure 5 in the manuscript

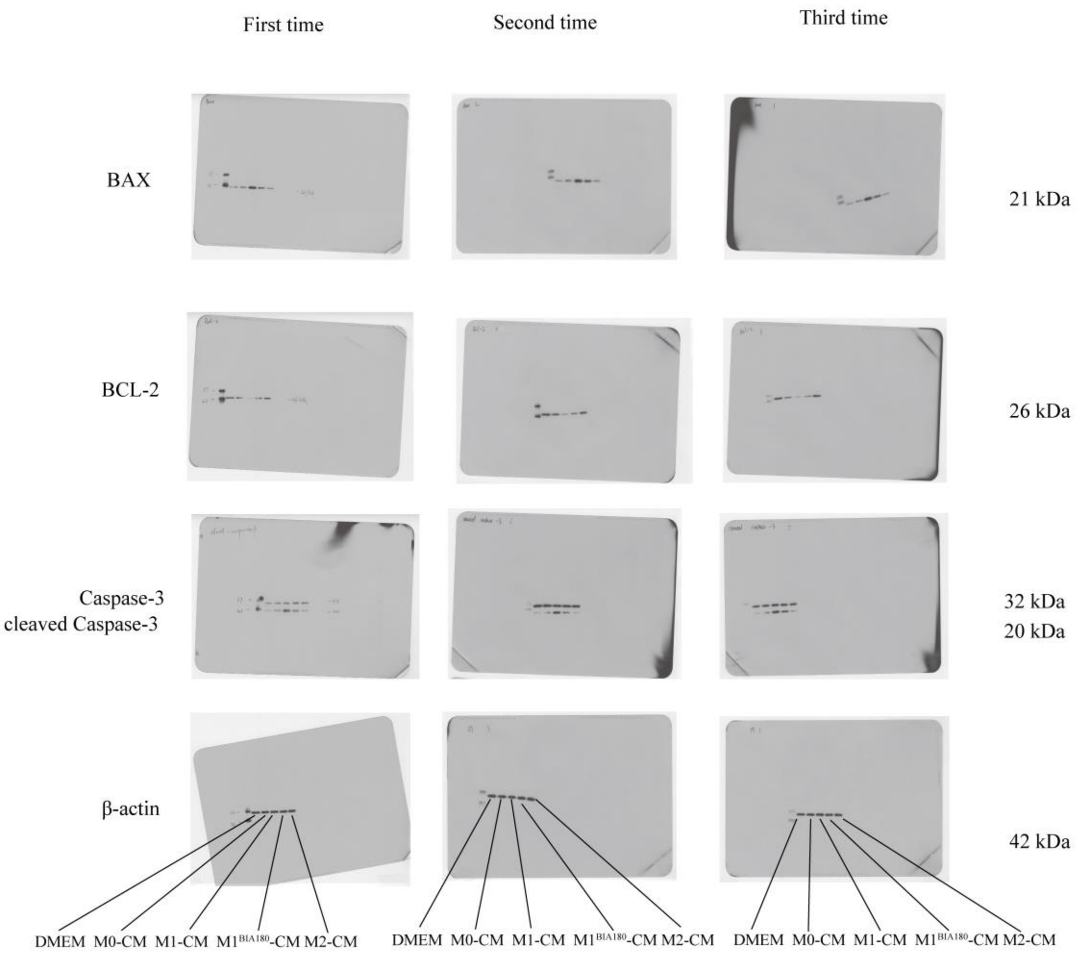

Original blots/gels of Figure S2

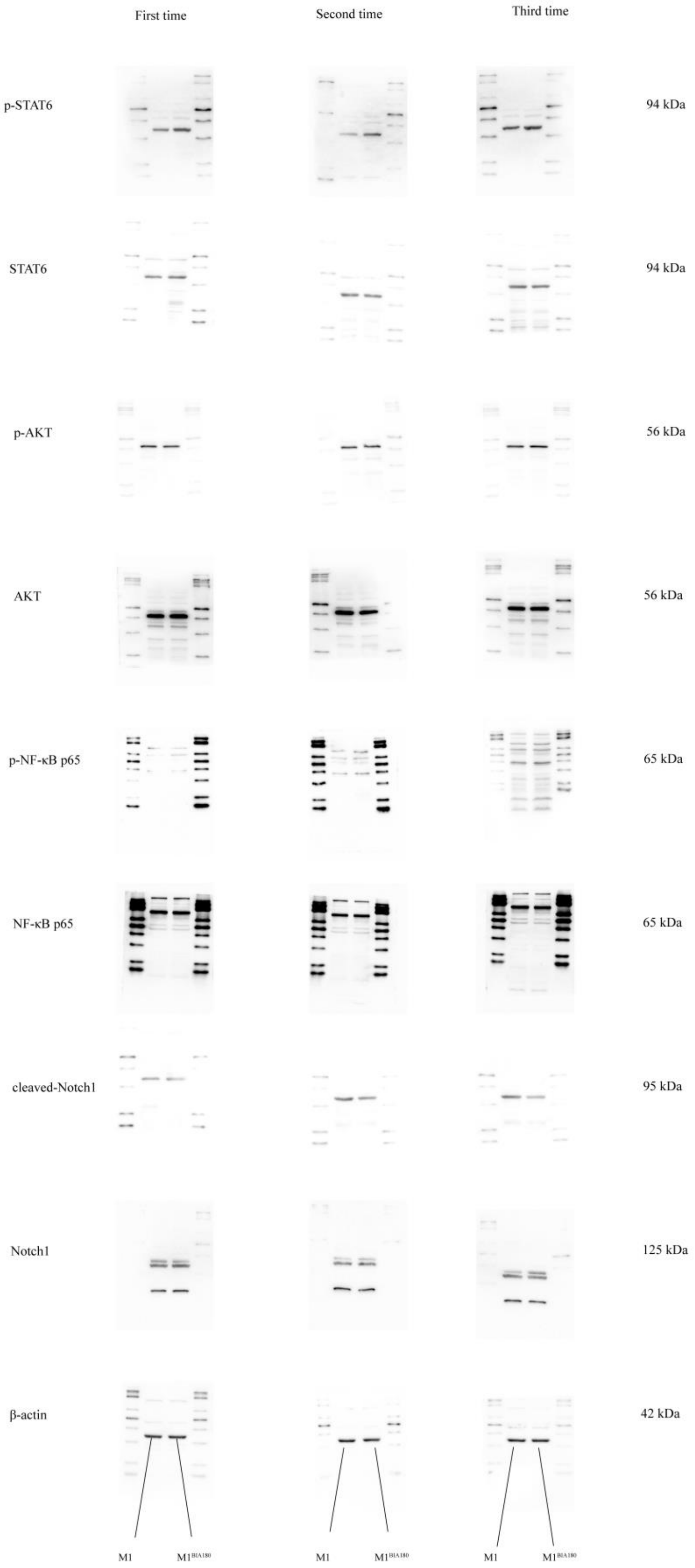

Supplement: Supplementary file 1 — Supplementary Material 1 [file 41598_2024_83198_MOESM1_ESM.pdf]
